# Assembly of complete diploid-phased chromosomes from draft genome sequences

**DOI:** 10.1093/g3journal/jkac143

**Published:** 2022-06-10

**Authors:** Andrea Minio, Noé Cochetel, Amanda M Vondras, Mélanie Massonnet, Dario Cantu

**Affiliations:** Department of Viticulture and Enology, University of California Davis, Davis, CA 95616, USA; Department of Viticulture and Enology, University of California Davis, Davis, CA 95616, USA; Department of Viticulture and Enology, University of California Davis, Davis, CA 95616, USA; Department of Viticulture and Enology, University of California Davis, Davis, CA 95616, USA; Department of Viticulture and Enology, University of California Davis, Davis, CA 95616, USA

**Keywords:** haplotype phasing, diploid genomes, assembly error correction, hybrid genome assembly, chromosome anchoring

## Abstract

*De novo* genome assembly is essential for genomic research. High-quality genomes assembled into phased pseudomolecules are challenging to produce and often contain assembly errors because of repeats, heterozygosity, or the chosen assembly strategy. Although algorithms that produce partially phased assemblies exist, haploid draft assemblies that may lack biological information remain favored because they are easier to generate and use. We developed HaploSync, a suite of tools that produces fully phased, chromosome-scale diploid genome assemblies, and performs extensive quality control to limit assembly artifacts. HaploSync scaffolds sequences from a draft diploid assembly into phased pseudomolecules guided by a genetic map and/or the genome of a closely related species. HaploSync generates a report that visualizes the relationships between current and legacy sequences, for both haplotypes, and displays their gene and marker content. This quality control helps the user identify misassemblies and guides Haplosync’s correction of scaffolding errors. Finally, HaploSync fills assembly gaps with unplaced sequences and resolves collapsed homozygous regions. In a series of plant, fungal, and animal kingdom case studies, we demonstrate that HaploSync efficiently increases the assembly contiguity of phased chromosomes, improves completeness by filling gaps, corrects scaffolding, and correctly phases highly heterozygous, complex regions.

## Introduction

Affordable high-throughput DNA sequencing and novel assembly tools have made high-quality genome assemblies and genome research attainable and abundant. Long-read DNA sequencing technologies, like those developed by Oxford Nanopore Technologies and Pacific Biosciences, are now the preferred methods for reference genome sequencing. The assemblies produced using these technologies are more contiguous and complete than assemblies constructed using short sequencing reads and better represent repetitive content (Rhoads and Au 2015; Bongartz and Schloissnig 2019; Du and Liang 2019; Paajanen *et al.* 2019). Another important advantage of long-read sequencing is the ability to generate phased diploid assemblies. Previously, genome complexity due to heterozygosity was typically handled by generating a haploid representation of a diploid genome either by collapsing heterozygous sites into a consensus sequence or by including only 1 allele’s sequence (Small *et al.* 2007; Huang *et al.* 2012; Di Genova *et al.* 2014; Hirakawa *et al.* 2014; Kajitani *et al.* 2014; Ying *et al.* 2019).

Partially phased assemblies have revealed genomic complexities that were inaccessible in previous haploid representations, such as haplotype-specific structural variation events, trait-associated alleles, and allele-specific gene expression and methylation (Garg *et al.* 2021; Low *et al.* 2020; Massonnet *et al.* 2020; Sun *et al.* 2020; Zhou *et al.* 2020; Mansfeld *et al.* 2021). However, phasing diploid assemblies remains challenging for complex genomes. High heterozygosity and repetitive content often prevent phasing in diploid regions. This inflates the primary assembly (Chin *et al.* 2016; Minio *et al.* 2019) and can impair scaffolding procedures that use the primary assembly as input.

Hybrid approaches that integrate additional independent data, such as optical maps or chromatin structure, help scaffold draft genome assemblies up to full-length chromosomes (Barchi *et al.* 2019; Hosmani *et al.* 2019; Wallberg *et al.* 2019; Miga *et al.* 2020). Several genetic map-based and reference-guided scaffolding tools have been developed (Kim *et al.* 2013; Tang *et al.* 2015; Tamazian *et al.* 2016; Alonge *et al.* 2019). However, these tools assemble one haplotype at a time and do not make use of the information of the alternative haplotype (e.g. sequence homology, shared genetic markers, orthologous genes, primary-to-haplotig relationship) to aid the reconstruction and phasing of each haplotype. Consequently, constructing chromosome-scale pseudomolecules using these tools relies on the phasing accuracy of the draft genome, the density of genetic map markers, or similarity to a related species’ genome (Ren *et al.* 2012; Tang *et al.* 2015; Alonge *et al.* 2019). Though quality control is an integral part of the assembly procedure, the relationship between haplotypes is never included in quality control processes.

Here, we present HaploSync, an open-source package that scaffolds, refines, and fully phases diploid and chromosome-anchored genomes. HaploSync leverages the relationship between haplotypes to improve the quality and accuracy of assemblies, separates haplotypes while reconstructing chromosome-scale pseudomolecule sequences, and recovers a location for genomic regions that cannot be placed during other assembly steps. Quality controls are implemented at each step to check for and correct assembly errors. HaploSync was benchmarked using 5 diploid species with different levels of heterozygosity from the plant, animal, and fungal kingdoms. For each species, HaploSync delivered a completely phased, chromosome-scaled genome with a quality comparable to the assemblies considered as references for each species. HaploSync, its manual, and tutorials for its use are freely available at https://github.com/andreaminio/haplosync.

## Materials and methods

**Fig. 1.**
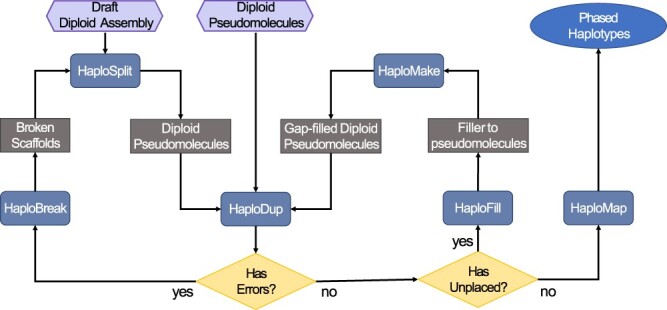
The HaploSync pipeline builds and refines haploid and diploid genome assemblies. The diploid-aware pipeline can deliver fully phased diploid pseudomolecules using a draft diploid assembly or diploid pseudomolecules as input. If draft sequences are used, Haplosplit first separates the haplotypes into 2 pseudomolecule sets. Pseudomolecules provided by the user or reconstructed with HaploSplit, then undergo quality control with HaploDup. If errors are found, input sequences can be edited with HaploBreak prior to rebuilding the pseudomolecules with HaploSplit. If no errors are detected and there are unplaced sequences, the pseudomolecule undergoes gap-filling with HaploFill. After each filling iteration, quality control can be performed with HaploDup. Finally, HaploMap can be used to identify colinear regions between pseudomolecules.

HaploSync has 6 modules: HaploSplit, HaploDup, HaploBreak, HaploFill, HaploMake, and HaploMap. The overall HaploSync workflow is summarized in Fig. 1. HaploSync accepts draft genome sequences or assembled pseudomolecules as input, preferably with minimally collapsed heterozygous sequences and no haploid consensus sequences. Allele phasing is unnecessary *a priori*. The tool is applicable to conventional haploid and diploid-aware assemblies.

### HaploSplit

HaploSplit uses external information to associate draft assembly sequences with original chromosomes, then sorts and orients them in pseudomolecules using directed adjacency networks. Alternative sequences are detected and segregated in 2 different haplotypes and, if the external information relates to a chromosome, HaploSplit delivers chromosome-scale scaffolds.

**Fig. 2.**
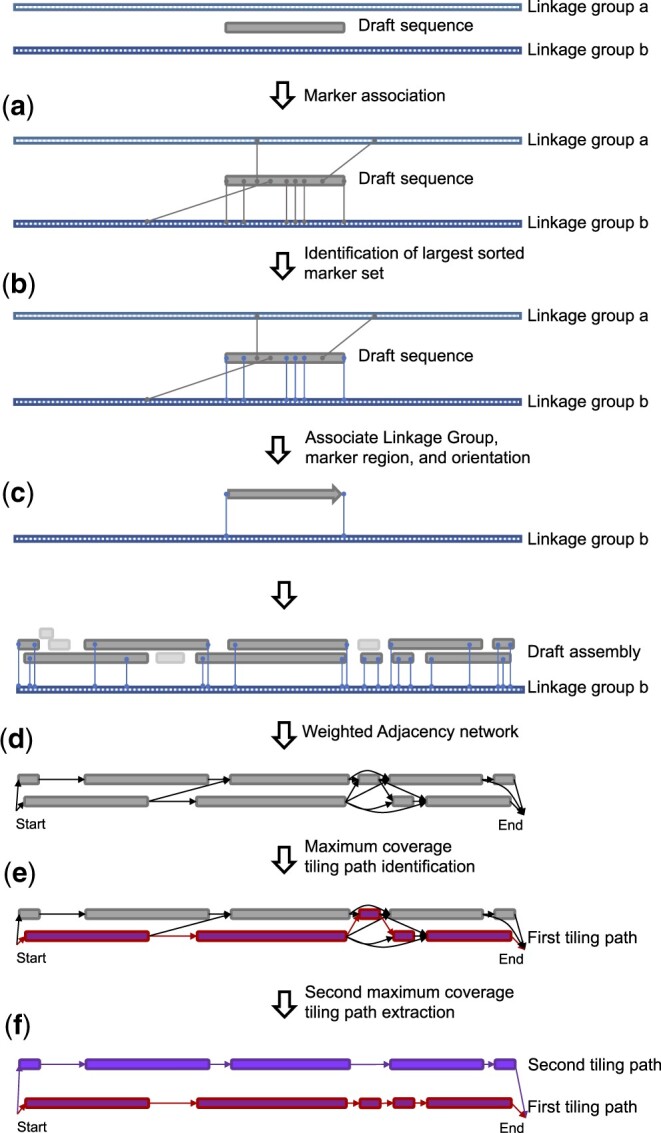
The HaploSplit procedure using genetic markers as input. a) The procedure identifies marker positions in the draft sequences. b) The longest sorted set of markers is identified for each draft sequence. c) Each sequence is assigned to a unique genomic region in the map (linkage group) and oriented. d) A directed adjacency network of nonoverlapping sequences is built for each linkage group connecting all sequences with no overlapping ranges of genetic markers. Sequences sharing markers are placed in separate network paths. e) The tiling path that maximizes the number of covered markers is selected for the first haplotype. f) Sequences belonging to the first haplotype are removed from the adjacency network and the second-best tiling path is used to scaffold the second haplotype.

External information can be a genetic map composed of sorted unique genomic markers (Fig.2) and/or the genome assembly of a closely related species (Supplementary Fig.1). When both types of information are used in hybrid mode, the genetic map is used as primary information to generate draft diploid pseudomolecules. The guide genome is used subsequently when marker information is insufficient. Phasing information between the alternative alleles is not needed *a priori*; HaploSplit will detect the existing relationship between haplotypes and phase them. The tool is capable of handling diploid assemblies lacking phasing information as well as diploid assemblies with inflated primary assemblies due to erroneous phasing. However, if the relationship between input sequences is known, it can be supplied to HaploSplit as a constraint to guide the reconstruction. For example, allelic information can be given to avoid placing primary contigs and haplotigs in the same haplotype.

If a genetic map is given as external evidence, HaploSplit first assesses markers’ uniqueness and congruence in the assembly. Markers present at 3 or more locations in draft sequences and markers present twice in the same draft sequence are considered unreliable and are excluded from further analysis. For each sequence containing an unreliable marker, HaploSplit produces a report containing layered interactive plots (Supplementary Fig. 2), including the sequence’s self-alignment, the position of reliable and duplicated genetic markers, and the copy number of annotated genes if gene annotation is available. If the input draft sequence is a scaffold, its composition in terms of legacy contigs is also included. These plots can be used to investigate the source of marker duplication within a draft sequence and to correct it using either HaploBreak (see below) or a constraint file. After identifying the genetic markers that are reliable for scaffolding, HaploSplit assigns draft sequences to a chromosome based on their largest set of consecutive markers (Fig. 2b), with their orientation based on markers’ order (Fig. 2c). If marker order does not adequately define sequence orientation (e.g. only 1 marker is present), the sequence is aligned and oriented based on the alternative haplotype (i.e. the sequence sharing the same marker). Once each draft sequence is assigned unambiguously to a chromosome, a directed, weighted adjacency network is created for each chromosome (Fig. 2d). Directed edges are created for each draft sequence with a weight based on the number of markers composing the sequence. Directed edges with zero weight are created to connect sequences without any common genetic marker ranges. Then, 2 haplotypes for each genomic region are split into different network paths. The tiling path that maximizes the number of genetic markers is used to scaffold the first haplotype and its draft sequences are removed from the adjacency network (Fig. 2e). The second-best tiling path is selected from the remaining sequences in the network and is scaffolded into the second haplotype (Fig. 2f).

If a genome is used to guide scaffolding (Supplementary Fig. 1), draft sequences are aligned on all guide genome sequences with Minimap2 (Li 2018). Local alignments are used to generate a directed weighted adjacency network for the query draft sequence and each guide genome sequence. Each draft sequence is associated with the guide sequence with which it shares the highest identity. Directed edges are created for each draft sequence with a significant alignment on the guide sequence. Directed edges between nonoverlapping hits are added to the network and connected with a weight of zero. For each adjacency network, the tiling path maximizing the number of matching bases between the draft sequences and the guide sequence is used to build the first haplotype. The second haplotype is then scaffolded using the second best path. As a consequence of the similarity-based scoring, haplotypes are built by combining the draft sequences with the highest homology to the guide genome at the risk of creating haplotype switches or over-fitting the guide genome's structure.

When a genetic map and a guide genome are used in hybrid mode, the genetic map is used as primary information to generate draft diploid pseudomolecules (Supplementary Fig. 1). The draft pseudomolecules and unplaced draft sequences are aligned to the guide genome. Then, an adjacency network is created for each guide sequence using the draft sequences composing each draft pseudomolecule and the unplaced draft sequences that do not significantly overlap the alignment of the draft pseudomolecules. The 2 tiling paths with the highest identity with the guide sequence are used for scaffolding the 2 haplotypes.

HaploSplit permits diverse, user-defined relationships between sequences to constrain and/or fine-tune scaffolding. For example, the relationship between the haplotigs and primary sequence defined by a sequence assembler like Falcon Unzip can be used to maintain consistency across alternative sequences. Similarly, a list of sequences in specific linkage groups can be given to guide their placement in pseudomolecule scaffolds.

### HaploDup

**Fig. 3.**
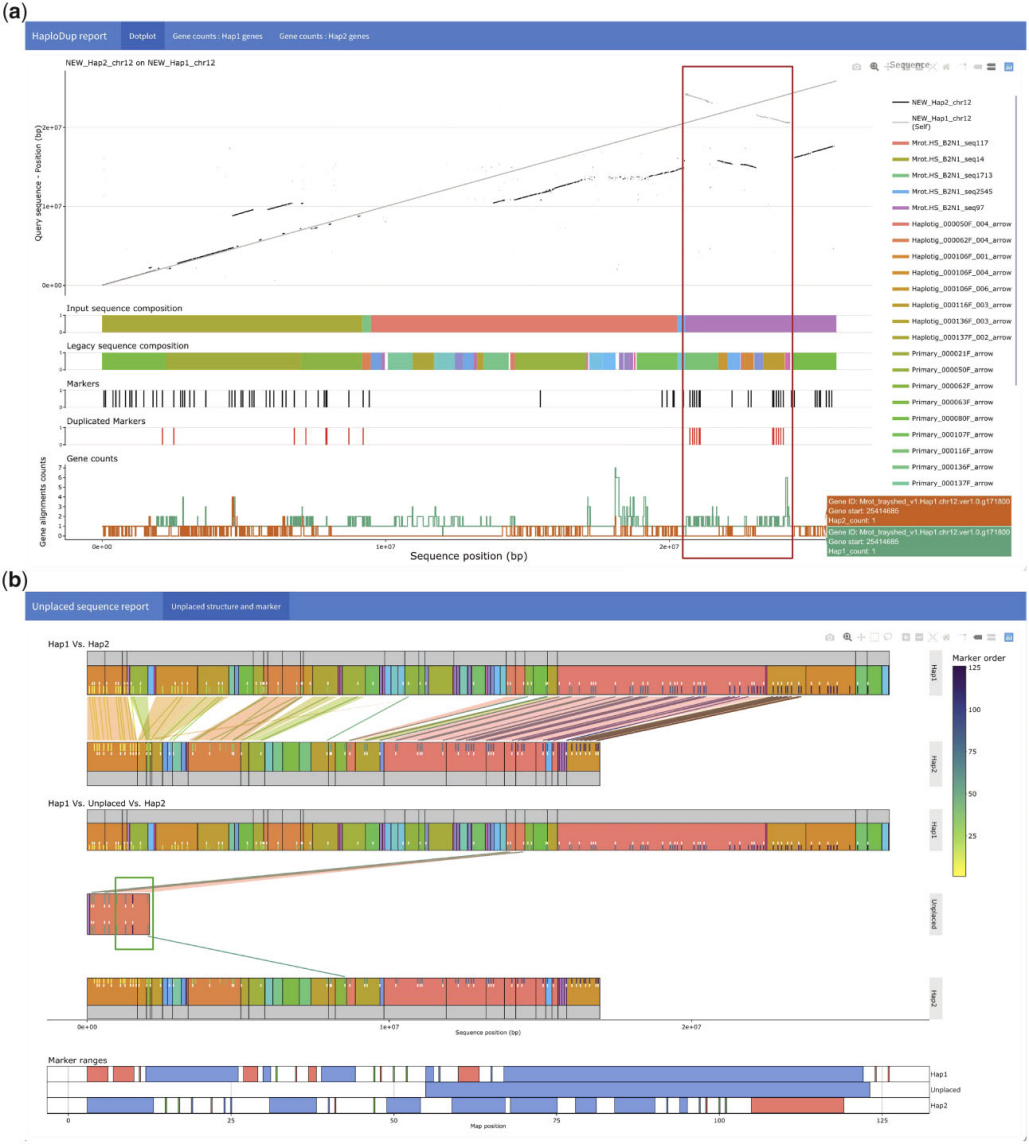
Example of HaploDup’s interactive reports. The figure reports 2 static screenshots exemplifying HaploDup interactive output. a) Assembly quality control of *M. rotundifolia* chromosome 12 Haplotype 1: whole-sequence alignment of both alternative haplotypes on Haplotype 1, legacy contig and hybrid scaffold composition of Haplotype 1, position of the genetic markers and the duplicated markers in Haplotype 1, number of significant alignment(s) per gene of Haplotype 1 in each alternative haplotype. In this example, the composition in legacy contigs and position of duplicated markers indicate that both alleles (primary contig and haplotig) and both marker copies were placed in a hybrid scaffold (overlayed box). b) Unplaced sequence quality control: Marker content is compared between pseudomolecules and unplaced sequences to evaluate conditions that prevent the inclusion of a specific unplaced sequence. Color-coding is used for better contextualization. Markers are color-coded based on their order in the map. The structure of pseudomolecules and unplaced sequences are represented with color-coded blocks. Blocks identify the composition in terms of draft assembly sequences, color coding is used to show the existing relationships between the composing sequences (e.g. primary to haplotig relationships). In this example, the presence of a marker (overlayed box, the dark marker on the right of the contig) in the unplaced sequences far from its expected position on the map extends the expected coverage of the map to the end of the linkage group and prevents placement in any haplotype scaffold.

HaploDup (Fig. 3 and Supplementary Fig. 3) exerts diploid-aware quality control over pseudomolecule sequences. HaploDup generates multiple sets of interactive plots that allow the user to identify misassemblies and expose conflicts that prevent correct sequence placement. Misassemblies can be caused by erroneous hybrid scaffolding (Supplementary Figs. 2–4), a lack of colinearity information with the guide genome (Supplementary Fig. 5), or incorrectly sorted genetic markers (Supplementary Fig.6). Misassemblies can be inherited by downstream assembly steps if not corrected (Fig. 3a).

To identify misassemblies and help plan a correction strategy, HaploDup compares pseudomolecule sequences, integrates structural (e.g. contigs and scaffolds) and feature (e.g. markers and genes) information, and produces interactive plots (Fig. 3). Two kinds of plots are generated. The first compares alternative haplotypes (Fig. 3a). The second visualizes unplaced sequences with sufficient information to be placed but are currently unplaced among scaffolds because of incompatibility with other sequences; these are compared with the 2 alternative pseudomolecules (Fig. 3b).

#### Alternative haplotype comparison

HaploDup produces a report for each alternative haplotype of each linkage group (Fig. 3a). The report includes layered plots: (1) alignment of the 2 alternative haplotypes on the target haplotype; (2) the target sequence structure, with 2 lines of sequences at most (if available); (3) marker position and duplication status (if available).

The dotplot is essential for visualizing colinear regions within and between pseudomolecules. Duplications, deletions, and translocations can be spotted by overlaying both haplotypes’ alignments. If this information is intersected with the structure of input contigs or scaffolds, then it is possible to determine whether these peculiarities are real or are technical errors. For example, a region duplicated in 1 haplotype and deleted in the other may indicate that both alleles were placed in the same scaffold instead of one placed in each haplotype (red box in Fig. 3). Genetic markers and genes’ positions also help identify assembly errors. Genetic markers that are duplicated within the genome assembly are indicative of misplaced alleles. When a gene annotation is available, HaploDup counts significant alignments (>80% coverage and identity) of each coding sequence (CDS) on its pseudomolecule of origin and on the alternative haplotype. This is useful for spotting fused haplotypes when the whole genome dotplot lacks resolution. An unbalanced number of gene copies between haplotypes in a given region can indicate a deficit of information or a duplication error. With these plots, the user can identify misassembled regions. Misassemblies can be solved by providing either a list of the breakpoint coordinates of the misplaced sequences to HaploBreak or a constraint file to HaploSplit.

#### Comparison of unplaced sequences with the 2 haplotypes of each pseudomolecule

HaploDup uses external information to compare unplaced sequences to related pseudomolecules (Fig. 3b). The plot reports: (1) a comparison of associated pseudomolecules structures in terms of markers and sequence content. Structure is reported on 2 levels (scaffolds input to HaploSplit and their composition in terms of legacy contigs) when the requisite information is available; (2) a comparison of the unplaced sequence to the associated pseudomolecules in terms of markers and sequence content at 2 levels (scaffolds input to HaploSplit and their composition in terms of legacy contigs); (3) a comparison of the ranges of markers covered by the unplaced sequence and the ranges covered by the draft sequences composing the pseudomolecules.

Markers and their relationship to sequences can be visualized. Markers can be color-coded based on order. This plot helps resolve conflicts that prevent sequence placement into linkage groups. In Fig. 3b and Supplementary Fig. 6, for example, a distal marker is incorrectly ordered inside an unplaced sequence. This triggered its exclusion from any of the pseudomolecules. Once fixed, the sequence will be placed.

### HaploBreak

HaploBreak (Supplementary Fig. 7) automatically searches for and breaks sequences at the nearest known junction or at the nearest gap. The coordinates of breakpoint pairs are given by the user to estimate where sequences should be broken to correct scaffolding errors. If a scaffolding structure is supplied by the user, these junctions are prioritized to be broken. If a pair of breakpoints leads to 2 distinct scaffolding junctions, the original sequences reported between the 2 junctions will be excluded from the tiling path. If either breakpoint in a pair is associated with a sequence instead of a junction, the corresponding original sequence is broken on the nearest gap (i.e. stretch of “N” characters between 2 contigs). For each pair of breakpoint coordinates queried by the user, HaploBreak will do the following procedure: (1) search for scaffolding junctions closest to the 2 coordinates. If a junction is found within the defined search limits, it is associated with the breakpoint, else the original sequences are searched for the closest gap (i.e. a region of “N” characters), (2) break the sequence. If the pair of coordinates is associated with 2 distinct scaffolding junctions (or 1 junction and the end of an input sequence), the original sequence between them is classified as misplaced (i.e. “unwanted” in that tiling path). If one or both breakpoints are associated with a gap in the original sequence, the sequence is broken at the gap position.

### HaploFill

A reference-independent approach, HaploFill (Supplementary Fig. 8) uses the relationship between homologous pseudomolecule scaffolds to improve the assembly’s completeness by integrating unplaced sequences where scaffolding gaps occur. Gaps are created during scaffolding procedures when adjacent regions in the pseudomolecule are assembled in separate sequences and lack sufficient information to connect them. Instead, a gap (i.e. stretches of “N” characters) is inserted as placeholder. When multiple scaffolding procedures are performed, gaps defined in previous iterations are inherited in the subsequent steps. HaploFill uses several reference-independent strategies to identify the specific kind of gap and the correct filler sequences.

A gap in a scaffold may occur when there is insufficient reliable information to identify the correct sequence for the region. This can happen when there is a lack of digestion sites in optical maps, a shortage of markers for HaploSplit, or when multiple alternative sequences are linked with proximity ligation data (e.g. mate-pair library, HiC libraries). A gap may also occur in a scaffold when the sequence is unavailable for placement. This can occur if it was not assembled or if one consensus sequence was produced from multiple genomic loci (e.g. repeats). This might also happen in diploid assemblies at homozygous regions where no alternative sequence is produced.

HaploFill is designed to recover gap information by comparing the gap region to the sequence present in the alternative haplotype. First, unplaced sequences are searched for the missing constituent. If no suitable candidate is found, the gap is filled using the alternative allele’s sequence.

HaploFill does the following steps. First, HaploFill will try to determine the ploidy of each region using sequencing coverage information: (1) align long or short sequencing reads onto each haplotype separately and calculate the base coverage along each pseudomolecule using Bedtools (Quinlan and Hall 2010); (2) calculate the expected haploid depth of coverage with a Savitzky–Golay filter for each pseudomolecule, excluding annotated repetitive regions; (3) classify each region of the genome as uncovered, haploid, diploid, and repetitive based on the ratio between the depth of coverage and the expected haploid depth of coverage. Thresholds can be defined by the user. For each gap, HaploFill extracts the region upstream and downstream of the gap and the corresponding regions on the alternative haplotype to build support sequences that will assist the search for filler.

If the alternative region is reliably diploid (i.e. neither repetitive nor extensively gapped on the opposite haplotype) HaploFill will (1) create a hybrid support sequence made of the regions flanking the gap and the regions corresponding to the gap on the alternative haplotype, (2) create an alternative support region made of the regions that correspond to and flank the gap on the alternative haplotype. If the region that corresponds to the gap on the alternative haplotype is highly repetitive or gapped, HaploFill will create 2 alternative support sequences made of the regions flanking the gap on the 2 haplotypes.

HaploFill will then search for gap filler among the unplaced sequences. To do this, HaploFill will first map unplaced sequences onto all the support regions with Nucmer (Marçais *et al.* 2018). Unplaced sequences are assigned globally in a 1-to-1 relationship to supporting sequences. Pairings are ranked based on the bases that match nonrepetitive portions of the support sequence and the whole support sequence. Then, the best filler is assigned to the gap. Filler priority is given to the hybrid support region filler, followed by the alternative support region, and then to the gapped support regions. If no filler can be validated to cover the gap but the corresponding region is classified as diploid based on sequencing coverage, the region is assumed to be homozygous. In this scenario, the region on the alternative haplotype corresponding to the gap is used as a filler. Like HaploSplit, HaploFill allows a wide range of user-defined relationships between sequences to fine tune the filler selection procedure. For example, the relationship between the primary and haplotigs can be used to consistently place alternative sequences.

### HaploMake

HaploMake automates the conversion of sequences and annotations between different assembly versions. As input, it accepts the FASTA of the genome and a structural file (e.g. AGP files, BED, and HaploFill output files) that describes the new sequence configuration. If a gene annotation, markers, or contig structures are given, HaploMake will automatically translate their coordinates relative to the new sequence. The ends of adjacent regions in the structure files can be checked for overlaps with Nucmer (Marçais *et al.* 2018). The coordinates of adjacent regions can be corrected by adjusting junction positions. This avoids duplicating genomic content in the final sequence and can be done without altering the gene annotation (Supplementary Fig. 9).

### HaploMap

HaploMap (Supplementary Fig. 10) performs a pairwise comparison between haplotypes and delivers a pairwise tiling map of colinear, nonoverlapping, and nonrepetitive regions between different haplotypes. Like HaploSplit, local alignments between each pair of sequences are performed with Minimap2 (Li 2018) or Nucmer (Marçais *et al.* 2018). Hits are used to create a weighted adjacency graph for identifying a bidirectional tiling path that maximizes the identity between the 2 sequences. The coordinates of the colinear regions that form the bidirectional tiling path are listed in a pairwise, phased map of matching sequences. Sequences are not modified by HaploMap.

### Testing datasets

HaploSync performance was tested using a wide range of species and assembly protocols (Table 1). The diploid *Candida albicans* draft assembly (Hamlin *et al.* 2019), built using PacBio reads and FalconUnzip (Chin *et al.* 2016), was anchored to chromosomes using the genetic map generated by Forche *et al.* (2004). A diploid genome assembly of *Arabidopsis thaliana* Columbia-0 (Col-0) × Cape Verde Islands (Cvi-0; Chin *et al.* 2016) was anchored using a genetic map from Singer *et al.* (2006). The *Bos taurus* Angus × Brahma genome from Koren *et al.* (2018) was assembled using FalconUnzip, anchored to chromosomes using the genetic map from Low *et al.* (2020), and integrated with sex chromosome information from the Integrated Bovine Map from Btau_4.0 release available from https://www.hgsc.bcm.edu/other-mammals/bovine-genome-project. To support the assembly and quality control of pseudomolecule reconstruction, the locations of unique genes from the respective reference annotations (*C. albicans* SC5314_A22, *A. thaliana* TAIR10, and *B. Taurus* Btau_ARS-UCD1.2) were identified by mapping CDS sequences on primary and haplotig sequences using GMAP (ver. 2019.09.12; Wu and Watanabe 2005). Unique gene models were defined by mapping CDS sequences from the reference genomes annotations on the respective reference genome sequences using GMAP (ver. 2019.09.12; Wu and Watanabe, 2005). All CDS mapping on multiple locations in the haploid genome were removed from the dataset. HaploFill was applied once to each of these 3 genomes. The *Vitis vinifera* ssp. *vinifera* cv. Cabernet Franc FPS clone 04 genome was assembled and scaffolded with PacBio reads and Dovetail HiC data (Vondras *et al.* 2021). *Muscadinia rotundifolia* cv. Trayshed contigs were assembled with FalconUnzip in hybrid scaffolds that used BioNano NGM maps (Cochetel *et al.* 2021). A *Vitis* consensus genetic map (Zou *et al.* 2020) was used to anchor both genomes to chromosomes in HaploSplit and followed by several iterations of HaploFill.

**Table 1.** Assembly statistics.

Genotype	Kingdom	Haploid size	Chromosomes	Technology	Markers (per Mb)	Input sequences		**Results** ^a^		
									HaploSplit	HaploFill
*C. albicans*	Fungi	14 Mb	7 + R	PacBio^b^	116 (8.3)^c^	Primary	15.5 Mb	Hap 1	11.6 Mb	12.9 Mb
						Haplotigs	13.8 Mb	Hap 2	12.4 Mb	13.7 Mb
						Total	29.2 Mb	Unpl	5.2 Mb	2.7 Mb
*A. thaliana*	Plantae	119 Mb	5	PacBio^d^	676 (5.7)^e^	Primary	140.0 Mb	Hap 1	109.0 Mb	114.7 Mb
						Haplotigs	104.9 Mb	Hap 2	106.6 Mb	111.5 Mb
						Total	245.0 Mb	Unpl	29.4 Mb	19.0 Mb
*B. taurus*	Animalia	2.6 Gb (29+X) 2.5 Gb (29 + Y)	29 + XY	PacBio^f^	46,325 (17.6)^g^	Primary	2.7 Gb	Hap 1	2.6 Gb (29+X)	2.6 Gb (29+X)
						Haplotigs	2.5 Gb	Hap 2	2.3 Gb (29+Y)	2.5 Gb (29+Y)
						Total	5.2 Gb	Unpl	0.3 Gb	0.2 Gb
*V. vinifera* cv. Cabernet Franc	Plantae	487–557 Mb^h^	19	PacBio + Doveatil HiC^i^	1,661 (3.5)^j^	Primary	570.2 Mb	Hap 1	350.8 Mb	455.6 Mb
						Haplotigs	284.7 Mb	Hap 2	263.4 Mb	410.9 Mb
						Total	854.9 Mb	Unpl	239.9 Mb	47.1 Mb
*M. rotundifolia*	Plantae	483 Mb	20	PacBio + BioNano^k^	1,661 (3.5)^l^	Primary	459.5 Mb^m^	Hap 1	374.3 Mb	400.5 Mb
						Haplotigs	364.8 Mb^n^	Hap 2	338.9 Mb	370.0 Mb
						Total	896.0 Mb	Unpl	165.5 Mb	63.0 Mb

Summary statistics for the testing dataset.

a Where Hap 1: Haplotype 1; Hap 2: Haplotype 2; Unpl: Unplaced sequences.

b FalconUnzip (Hamlin *et al.* 2019).

c Forche et al. (2004).

d FalconUnzip (Chin *et al.* 2016).

e Singer *et al.* (2006).

f FalconUnzip (Koren *et al.* 2018).

g Low et al. (2020) using the Integrated Bovine Map of sex chromosome (ver. Btau_4.0, https://www.hgsc.bcm.edu/other-mammals/bovine-genome-project).

h Range of values as reported for PN40024 in Jaillon *et al.* (2007) and Cabernet Sauvignon in Cochetel *et al.* (2021).

i FalconUnzip + SSPACE + HiRise (Vondras *et al.* 2021).

j Zou *et al.* (2020).

k FalconUnzip + Hybrid Scaffolder (Cochetel *et al.* 2021).

l Zou *et al.* (2020).

m Reported for FalconUnzip assembly as haplotype separation is lost during Hybrid Scaffolding.

n Reported for FalconUnzip assembly as haplotype separation is lost during Hybrid Scaffolding.

## Results and discussion

To evaluate HaploSync’s performance, 5 diploid species from 3 different kingdoms were selected. This included a *M.* *rotundifolia*, *V.* *vinifera*, and an F1 progeny of *A.* *thaliana* (Col-0 × Cvi-0; Chin *et al.* 2016), the bull *B. taurus* Angus × Brahma (Koren *et al.* 2018; Low *et al.* 2020), and pathogenic yeast *C.* *albicans*. These species are diverse and vary in genome size, chromosome number, repeat content, and amount of heterozygosity. Long sequencing reads, genetic maps, and public reference genomes are available for those species.

### HaploSync adaptability to different species

HaploSync produced high-quality genomes for all 5 species (Table 1). The resulting assemblies were nearly twice the size of their original haploid assemblies, with 1.87×–2.03× their gene space represented (Supplementary Table 1). This indicates that most of both haplotypes were assembled separately. High-density genetic maps and highly contiguous draft assemblies enabled HaploSplit to produce high-quality pseudomolecules that differed 5.8–17.8% from their expected chromosome sizes. In 1 iteration, HaploFill increased assembly completeness and reduced the difference in length between haplotypes.

For *C. albicans*, the limited number of markers was used to anchor 2.4 Mb of sequences to pseudomolecules in HaploSplit. The assembly had the highest share of unplaced sequences (5.2 Mb), but HaploFill recovered 17.9% of the missing genomic content in one iteration. The final pseudomolecules were up to 97.9% complete. Only 231 (3.8%) of 6,079 single-copy genes in the reference annotation mapping on the assembled sequences were not represented in the pseudomolecules produced by HaploSplit. This number was reduced to 182 (3.0%) in a single iteration of HaploFill. BUSCO analysis confirmed the nearly complete separation of alternative alleles with only 5 complete gene models found in multiple copies in Haplotype 1 (3 genes) and Haplotype 2 (2 genes; Supplementary Table 1).

With 18.6 ± 0.6 markers/Mb, the genetic map of *B. taurus* autosomal chromosomes was the most dense out of the species used in this study. HaploSplit produced pseudomolecules almost identical in size to the ARS-UCD1.2 genome assembly (Rosen *et al.* 2020), with Haplotype 1 pseudomolecules deviating by 0.7 ± 0.7% and Haplotype 2 by 6.7 ± 3.0% (Supplementary Table 2 and Supplementary Fig. 11). HaploFill inserted 151 Mb, mostly in Haplotype 2 pseudomolecules, reducing missing information in Haplotype 2 pseudomolecules to 1.4 ± 1.9% of ARS-UCD1.2 chromosome sizes. For sex chromosomes, only a genetic map of the X chromosome with low marker density was available (2.1 markers/Mb, assembly ver. Btau_4.0 available at https://www.hgsc.bcm.edu/other-mammals/bovine-genome-project). As a consequence, HaploSplit’s performance dropped. HaploSplit retrieved 79.8% of the expected 139 Mb X chromosome. However, HaploFill reduced missing information to 9.7% (Supplementary Table 2 and Supplementary Fig. 11). Without markers available, the length of the Y chromosome was only 11% of its expected size (4.5 Mb). The gene space was more complete in terms of single-copy reference genes. Only 7 of 57,974 single-copy CDSs mapping on the assembled sequences were not placed in the initial pseudomolecules produced with HaploSplit. This was reduced to 5 by HaploFill. BUSCO analysis confirmed the completeness and the separation of the alleles, with 92.5% complete gene models found in Haplotype 1 (1.3% in multiple copies) and 86.8% in Haplotype 2 (1.2% multiple copies; Supplementary Table 1).

In plants, the high level of polymorphism and structural variation between haplotypes make assembly and phasing challenging (Chin *et al.* 2016). The high level of heterozygosity in the *A. thaliana* accession used to test HaploSync is caused by sequence variation between its parents, Col-0 and Cvi-0. This led to a primary assembly 17% longer and haplotigs 11.8% shorter (Chin *et al.* 2016) than the haploid reference genome. After Haplosync, the 2 sets of pseudomolecules differed by 3.6% and 6.3% from the haploid reference genome size. This supports the tool’s ability to phase duplicated primary content between haplotypes. When gene space completeness was estimated using single-copy genes in the reference annotation, similar results were obtained. The amount of single-copy CDSs mapping on the assembled sequences represent the 99.7% of the entire dataset (34,741 out of 34,854). After HaploSplit, unplaced sequences included 1,966 putative loci (5.7%). Of these, 261 (1.1%) were missing from the pseudomolecules. HaploFill further increased the completeness of the pseudomolecules to include 98.1% and 95.8% of the gene space in the 2 haplotypes. This reduced the putative, single-copy CDS loci among unplaced sequences to only 123. Over 97% complete BUSCO gene models were complete in Haplotype 1 and the Haplotype 2, with only 1.3% and 1.5% in multiple copies, respectively (Supplementary Table 1).

*Vitis* species can be 12% heterozygous (Velasco *et al.* 2007). Assemblies of the species can exhibit extensive loss of phase between primary sequences and associated haplotigs (Chin *et al.* 2016; Roach *et al.* 2018; Minio *et al.* 2019; Vondras *et al.* 2019; Zhou *et al.* 2019). In Cabernet Franc, for example, the primary assembly is inflated by 18.8% and haplotigs are 40.7% shorter than the expected haploid genome size. HaploSync was able to overcome these limitations for both species and placed over 93.0% of the sequences in phased pseudomolecules that were no more than 9.8% different in size. HaploSplit also automatically placed and correctly phased the grape sex determining region (Massonnet *et al.* 2020) in *Muscadinia* and *Vitis* species. Using the unique CDS sequences from PN40024 as a reference for *Vitis* gene space, 1,233 (6.2%) of genes could not be placed in Cabernet Franc pseudomolecules with HaploSplit and 223 (1.4%) of genes could not be placed in *M. rotundifolia* pseudomolecules. This fraction of gene coding sequences could not be placed because of high fragmentation and low, uneven marker density that negatively affected pseudomolecule reconstruction performance. Several iterations of HaploFill reduced the number of unplaced CDSs to 0.3% for both genomes. This included 91 and 46 unique genes among unplaced sequences for Cabernet Franc and Trayshed, respectively. This highlights HaploFill’s ability to recover gene space information. Completeness and phasing of both Haplotypes was confirmed with BUSCO: 93% complete models in Haplotype 1 and 83% in Haplotype 2.

### HaploSync performance adaptability to different assembly procedures

HaploSync was applied to 2 grapes, *M. rotundifolia* cv. Trayshed (Cochetel *et al.* 2021) and *V. vinifera* cv. Cabernet Franc (Vondras *et al.* 2021), to assess its adaptability to genomes assemblies produced using different strategies. Although contigs were produced with PacBio data and FalconUnzip for both draft assemblies, Trayshed and Cabernet Franc were scaffolded with different technologies. *M. rotundifolia* underwent hybrid scaffolding with PacBio and a NGM map, which matches optical fingerprints of DNA molecules with assembled sequences digested in silico with the same enzyme. Gaps were introduced where there was a low density of digestion sites. Systematic errors were observed at highly repetitive and heterozygous regions, including the RUN1/RPV1 locus on chromosome 12 (Supplementary Fig. 2). The differential expansion of TIR-NBS-LRR genes between haplotypes (Cochetel *et al.* 2021) may have caused their fusion in the same scaffold. These issues affected 50 hybrid scaffolds (326.2 Mb), required correction, and were easily found with HaploDup. For Cabernet Franc, scaffolding was performed using HiC data that produced chimeric scaffolds due to the presence of diploid information in the primary assembly. Both haplotypes of 108 of scaffolds (449 Mb) were included in the same assembled sequence (Supplementary Fig. 4).

After scaffold correction, both genome assemblies were anchored to chromosomes using a *Vitis* consensus genetic map (Zou *et al.* 2020). Low specificity and marker density (3.5 markers/Mb) affected the construction of pseudomolecules by HaploSplit and negatively affected HaploSync’s performance. Cabernet Franc was most affected, with only 350.8 and 263.4 Mb placed on Haplotype 1 and Haplotype 2, respectively (i.e. 75% and 55% of the reference haploid genome). Unpleaceable sequences were nearly half of Cabernet Franc’s expected haploid genome size (240 Mb). Trayshed’s assembly was more complete; Haplotype 1 and Haplotype 2 assemblies were 374.3 and 338.8 Mb long, respectively.

Three iterations of HaploFill were performed on Cabernet Franc’s assembly. Each iteration reduced unplaced sequences by nearly one-half (Supplementary Fig. 12). The final Cabernet Franc pseudomolecules were 456 Mb (Haplotype 1) and a 411 Mb (Haplotype 2). Afterwards, 47 Mb (5.4%) of sequences remained unplaced. In contrast, only 2 iterations of HaploFill were sufficient to leave just 8% of Trayshed sequences unplaced. Haplotype 1 and Haplotype 2 of Trayshed’s pseudomolecules were 400 and 370 Mb, respectively. The total sizes of both haplotypes in both chromosome-scale assemblies were similar to their expected haploid reference genome sizes (Jaillon *et al.* 2007; Canaguier *et al.* 2017) and Cabernet Sauvignon’s haplotypes (459 and 449 Mb, respectively; Massonnet *et al.* 2020).

### HaploSync performance assessment

The performance of different HaploSync tools, in terms of result quality and processing time, is influenced by multiple factors. Unsurprisingly, the genome size and the number of linkage groups affect all assembly phases and the duration of alignment procedures. For HaploDup, HaploFill, HaploMap, HaploBreak, and HaploMake, genome size determines the size of the output and how long alignments take to complete, which can constitute over 90% of the computational time. The number of linkage groups exponentially increases the number of comparisons and plots needed. For example, HaploDup required 40 h to process *B. taurus*, which has a 2.6 Gb haploid genome size in 30 linkage groups and is the largest dataset used in this study. Nearly 15 of these hours were consumed by alignments between sequences while using 24 cores. *Candida* *albicans* is the smallest dataset, with 14 Mb in 8 linkage groups. In contrast to *B. taurus*, the same procedure required 75 min, with only 5 min dedicated to mapping.

HaploFill performance is also affected by the number of phased genomic sequences in the pseudomolecules. Alternative pseudomolecules are the backbone that enables the algorithm to retrieve gap filling information. The completeness of the pseudomolecules directly affects the amount of information usable as support for sequence placement. Unplaced sequences are information that might be recovered. The workflows adopted for *A. thaliana* and for the *Vitis* genotypes were selected based on pseudomolecule completeness. The *A. thaliana* assembly had relatively low sequence fragmentation and a high-density map. The pseudomolecules created for *A. thaliana* with HaploSplit were fairly complete after a single filling procedure. HaploSplit was less effective for Cabernet Franc and Trayshed because their assemblies were more fragmented and their maps were less dense. The workflow used for the grape genomes included several iterations of HaploFill to achieve highly complete pseudomolecules (Supplementary Fig. 12).

HaploSplit is fast. It takes between a few seconds and 1 min to build the adjacency graph, traverse it, find the 2 best tiling paths, and report the structure of the phased pseudomolecules. In contrast, the input quality control and the alignment between the draft sequences and the guide genome in preparation for the graph creation can be time-consuming. HaploSplit result quality is affected by several factors. The disparity and incomplete representation of both alternative alleles affect the completeness of the diploid pseudomolecules produced and necessitate filling. *A. thaliana* and *B. taurus* are F1 progeny. Their considerable structural variability is captured by the FalconUnzip assembler, which reconstructs the alleles fully and separately. In contrast, Cabernet Franc and Trayshed have several homozygous regions that were assembled in a single copy and highly heterozygous regions that increased the fragmentation of the contigs by fooling the assembler into overassembling the primary sequences. This difference is reflected in HaploSplit’s results. HaploSplit was able to separate alleles and deliver a nearly complete diploid assembly of *A. thaliana* and *B. taurus*. *Vitis* required a more extensive filling procedure to recover the missing information.

**Fig. 4.**
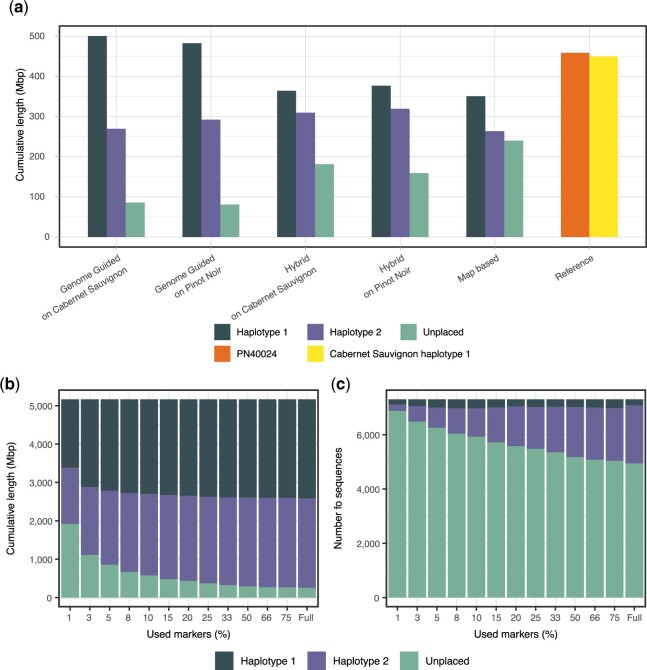
HaploSplit performance. a) The results of using different sources of external information and HaploSplit protocols for *V. vinifera* cv. Cabernet Franc cl. 04 (Vondras *et al.* 2021) assembly. Map-based assembly produces the largest first haplotype, but its overassembly occurs at the expense of the second haplotype’s completeness. A map-based approach is conservative and limited by the density of the markers. The hybrid approach recovers more sequences where the map is lacking information, without overassembling, and delivers a better reconstruction of both haplotypes. b) Effect of limited marker availability on overall assembly length tested on *B. taurus* Angus × Brahma (Koren *et al.* 2018; Low *et al.* 2020) by subsampling the genetic map. Longer sequences are more likely to contain a marker, making the first reconstructed haplotype most complete across all tests and with little variation in size. As the number of available markers increases and short sequences are included, the completeness of the second haplotype improves. c) Effect of limited marker availability on the number of placed sequences tested on *B. taurus* Angus × Brahma (Koren *et al.* 2018; Low *et al.* 2020) by subsampling the genetic map. Increasing the number of markers as fragmentation increases allows recruiting more sequences for scaffolding and improves completeness. Haplotype 1, with long sequences, shows little variation. In contrast, Haplotype 2 greatly benefits from increased marker density. The majority of sequences that remained unplaced are short and a small fraction of the genome’s length.

HaploSplit can use a genetic map and/or a guide-genome as information to facilitate scaffolding. We tested how HaploSplit performs given different scaffolding information using *V.* *vinifera* cv. Cabernet franc cl. 04 (Vondras *et al.* 2021). Reference genomes of closely related accessions, PN40024 (Canaguier *et al.* 2017) and Cabernet Sauvignon (Massonnet *et al.* 2020), are available. Cabernet Franc contigs were scaffolded (a) with the genetic map of Zou *et al.* (2020), (2) using the PN40024 V2 assembly or the first haplotype of Cabernet Sauvignon as guides, or (3) using both the genetic map and a guide genome. The reference-based approach incorporated more sequences into pseudomolecules than when only a genetic map was used. As expected, the best results were obtained using Cabernet Sauvignon as a reference, which shares 1 allele with Cabernet Franc. This approach, however, led to overfitting of the scaffolding results to the guide. Small structural variants in long draft sequences (Supplementary Fig. 13, A boxes) can find a proper representation thanks to neighboring colinear regions. Larger structural variants that encompass multiple sequences may fail to be reported correctly together. Each draft sequence location is identified independently from the others based on colinearity with guide genome, so placement is based on the structure of guide sequences rather than their actual order (Supplementary Fig. 13, B boxes). Moreover, gaps or the lack of information in the guide genome may impede the recovery of novel information. Only draft sequences that partially anchor within present information can be placed (Supplementary Fig. 13, C boxes). As a consequence, fragmented draft assemblies and the second haplotype are prone to be artificially similar to the guide genome. The hybrid approach performs better. The reconstruction of both haplotypes is more complete than the map-based approach, with the second haplotype benefiting most from this strategy (Fig. 4a). Though no overassembly was observed, the mapping phase duplicated some alleles. Both copies of several markers occurred in the same pseudomolecule scaffold when Cabernet Sauvignon (5 markers) and PN40024 (4 markers) were used as guides.

The effect of the number of reliable genetic markers on the performance of HaploSplit was tested on the genome of *B. taurus* Angus × Brahma (Koren *et al.* 2018; Low *et al.* 2020). The same diploid genome underwent chromosome-scale reconstruction using a randomly selected subset of 479 markers (1%, 0.2 markers/Mb) out of its available genetic map (46,323 markers, 17.6 markers/Mb; Fig. 4, b and c and Supplementary Fig. 14). Unsurprisingly, the number of unplaced sequences increased to 37% of the total assembly length given lower marker density. HaploSplit found a location in pseudomolecules for 99.4–100% of the sequences with markers. The performance of the algorithm, in terms of completeness of the delivered pseudomolecules, is primarily influenced by input assembly fragmentation and the genetic map’s marker density. This limits the number and the sizes of the sequences with markers. The primary assembly is composed of extremely long sequences that likely contain markers and are placed even when map density is low. In contrast, Haplotigs are more fragmented and require high marker density for comparable coverage. As a result, the first haplotype assembly is more complete even with fewer markers present (Fig. 4, b and c).

In summary, the type and quality of the external guide information have a large effect on the quality of the final assembly. A guide genome aids assembly via local sequence alignments; lack of homology between sequences and repetitive regions can cause segregation errors (Supplementary Fig. 6), misplacements, and overfitting to the guide genome structure. Genetic maps are more conservative, with the uniqueness of markers requiring a coherent placement within a map, if at all. Moreover, errors in the map can be more easily addressed by the user than errors in the guide genome sequence. The efficiency of HaploSync relies heavily on map precision (Supplementary Fig. 6) and the density and evenness of its markers (Table 1).

### Conclusions

These results emphasize the importance of controlling and correcting the sequences used as input to HaploSplit to prevent scaffolding errors. Although map quality and marker density affect pseudomolecule construction by HaploSplit, HaploFill generated phased assemblies with few unplaced sequences and sizes similar to their haploid reference genomes.

Sequencing technologies and assembly tools are continuously improving. HaploSync delivers assemblies with unprecedented quality and contiguity that can provide novel insight into genome structure and organization. The HaploSync suite of tools can be used to address some of the remaining impediments to genome reconstruction and improve assembly quality by taking advantage of diploid information that is readily available. HaploSync correctly and completely phases diploid genomes, reconstructs pseudomolecules by recovering missing information, and exerts quality control over the results.

## Web resources

HaploSync is freely available for download at GitHub https://github.com/andreaminio/haplosync. Instructions for installation, a full list of dependencies, a description of each tool, and tutorials are available in HaploSync’s manual (https://github.com/andreaminio/HaploSync/tree/master/manual).

## Data availability

The data used in this study are summarized in Supplementary Table 3. Pseudomolecule reconstructions of *C.* *albicans* NCYC4145 (Hamlin *et al.* 2019), *A.* *thaliana* Col-0 × Cvi-0 (Chin *et al.* 2016), and *B.* *taurus* Angus × Brahma (Koren *et al.* 2018) are available at Zenodo (https://zenodo.org/record/3987518, DOI: 10.5281/zenodo.3987518). *Vitis vinifera* cv. Cabernet Franc cl. 04 (Vondras *et al.* 2021) and *M.* *rotundifolia* cv. Trayshed (Cochetel *et al.* 2021) pseudomolecule assemblies are available at www.grapegenomics.com.

Supplemental material is available at *G3* online.

## Funding

This work was funded by the National Science Foundation (NSF) award #1741627 and the US Department of Agriculture (USDA)-National Institute of Food and Agriculture (NIFA) Specialty Crop Research Initiative award #2017-51181-26829. It was also partially supported by funds from E.&J. Gallo Winery and the Louis P. Martini Endowment in Viticulture.

## Conflicts of interest

None declared.

## Author contributions

AM and DC conceptualized the project. AM developed the methodology and software. AM and NC performed bioinformatic analyses and tested the software. AM, NC, and AMV wrote the software manual and pipeline walk-through. AM, AMV, MM, and DC wrote the article. DC secured the funding and supervised the project. All authors have read and approved the article.

## Supplementary Material

jkac143_Supplementary_Figure_1

jkac143_Supplementary_Figure_2

jkac143_Supplementary_Figure_3

jkac143_Supplementary_Figure_4

jkac143_Supplementary_Figure_5

jkac143_Supplementary_Figure_6

jkac143_Supplementary_Figure_7

jkac143_Supplementary_Figure_8

jkac143_Supplementary_Figure_9

jkac143_Supplementary_Figure_10

jkac143_Supplementary_Figure_11

jkac143_Supplementary_Figure_12

jkac143_Supplementary_Figure_13

jkac143_Supplementary_Figure_14

jkac143_Supplemental_Table_1

jkac143_Supplemental_Table_2

jkac143_Supplemental_Table_3
