# Supplementary figures and images for: Assembly of complete diploid-phased chromosomes from draft genome sequences
Source: G3 (Bethesda). 2022 Jun 10;12(8):jkac143. doi: 10.1093/g3journal/jkac143 (PMC9339290; doi:10.1093/g3journal/jkac143)

Guide genome based

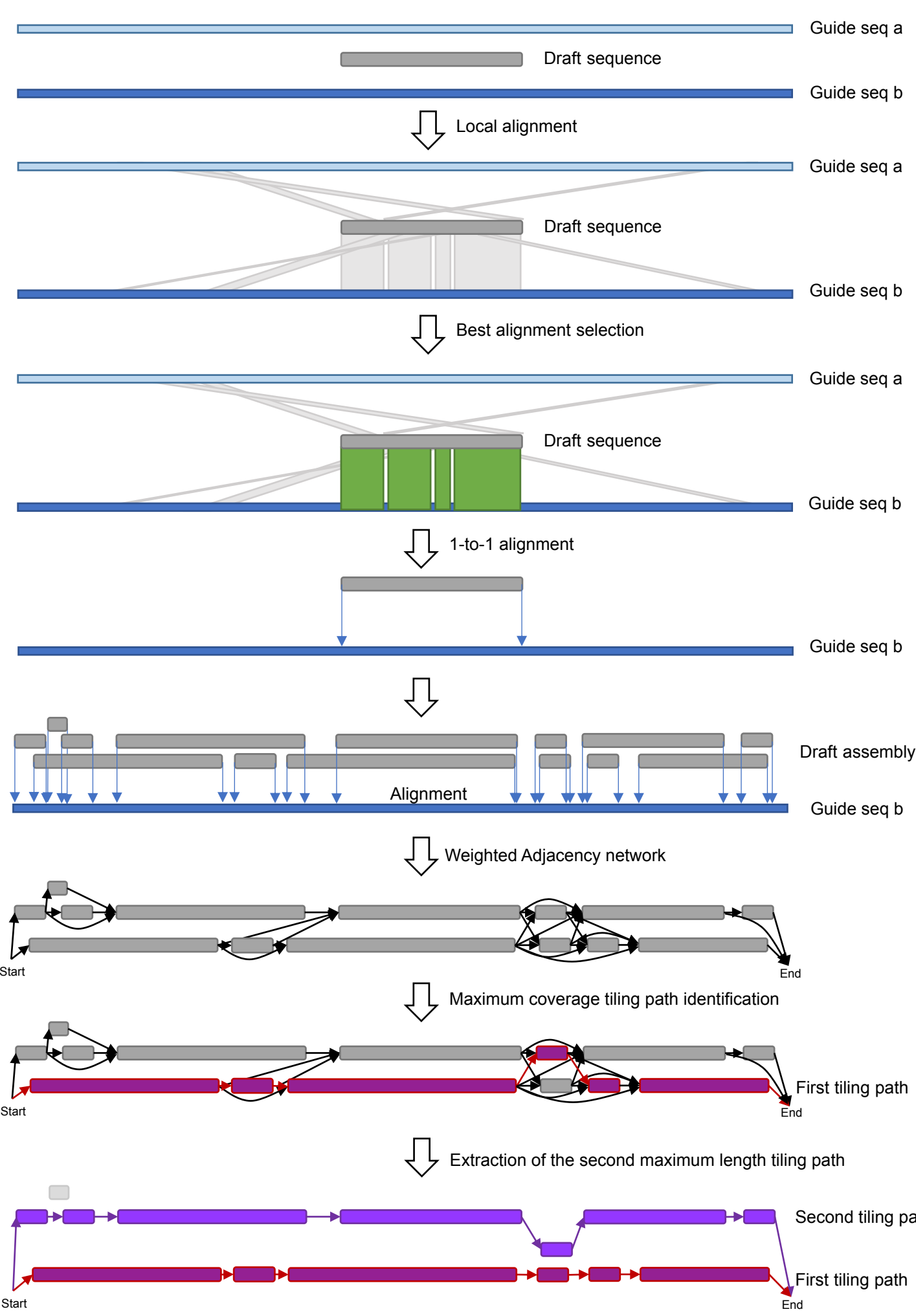

Hybrid genetic map and guide genome

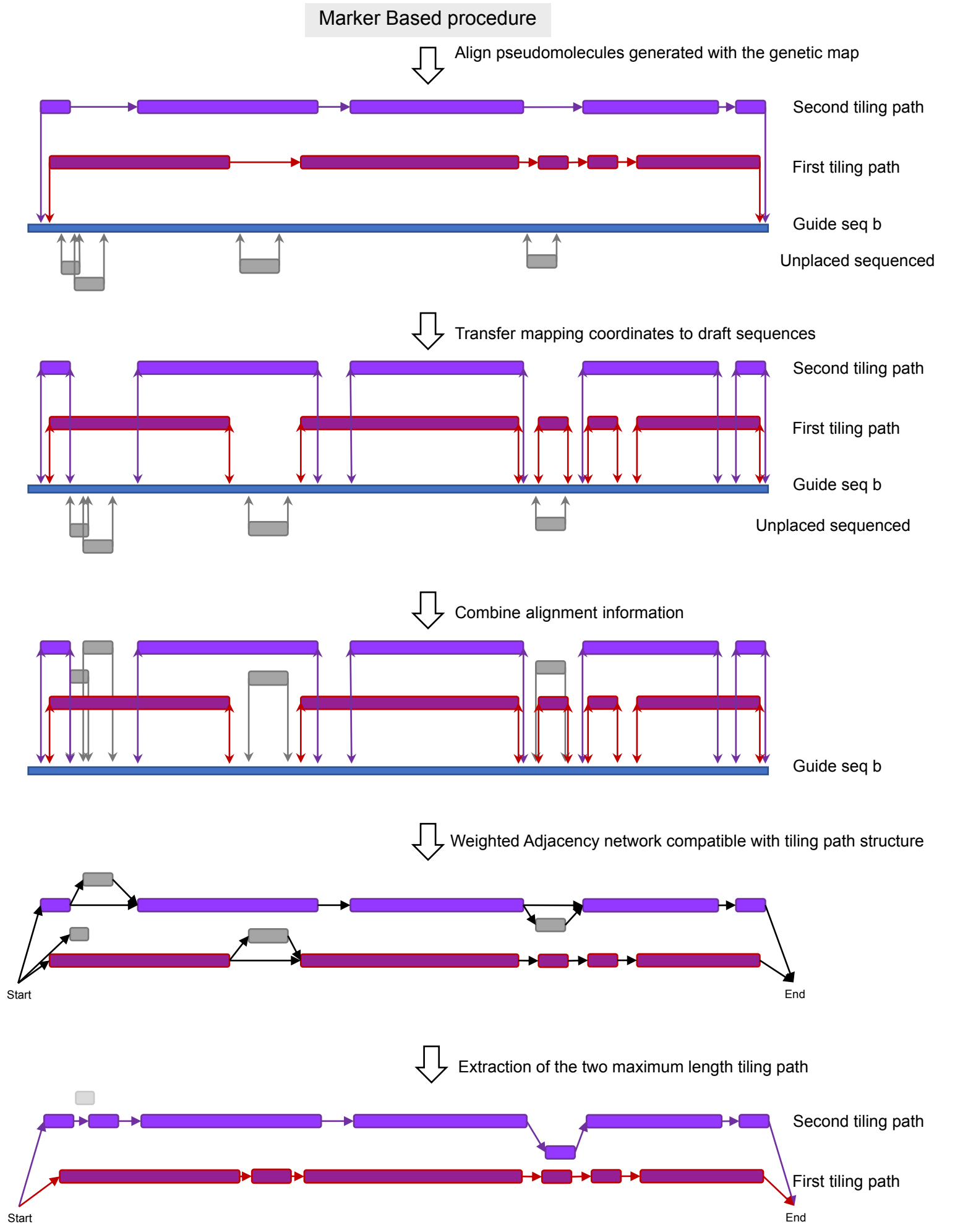

Supplement: jkac143_Supplementary_Figure_1 [file jkac143_supplementary_figure_1.pdf]

Intra-sequence duplication info report

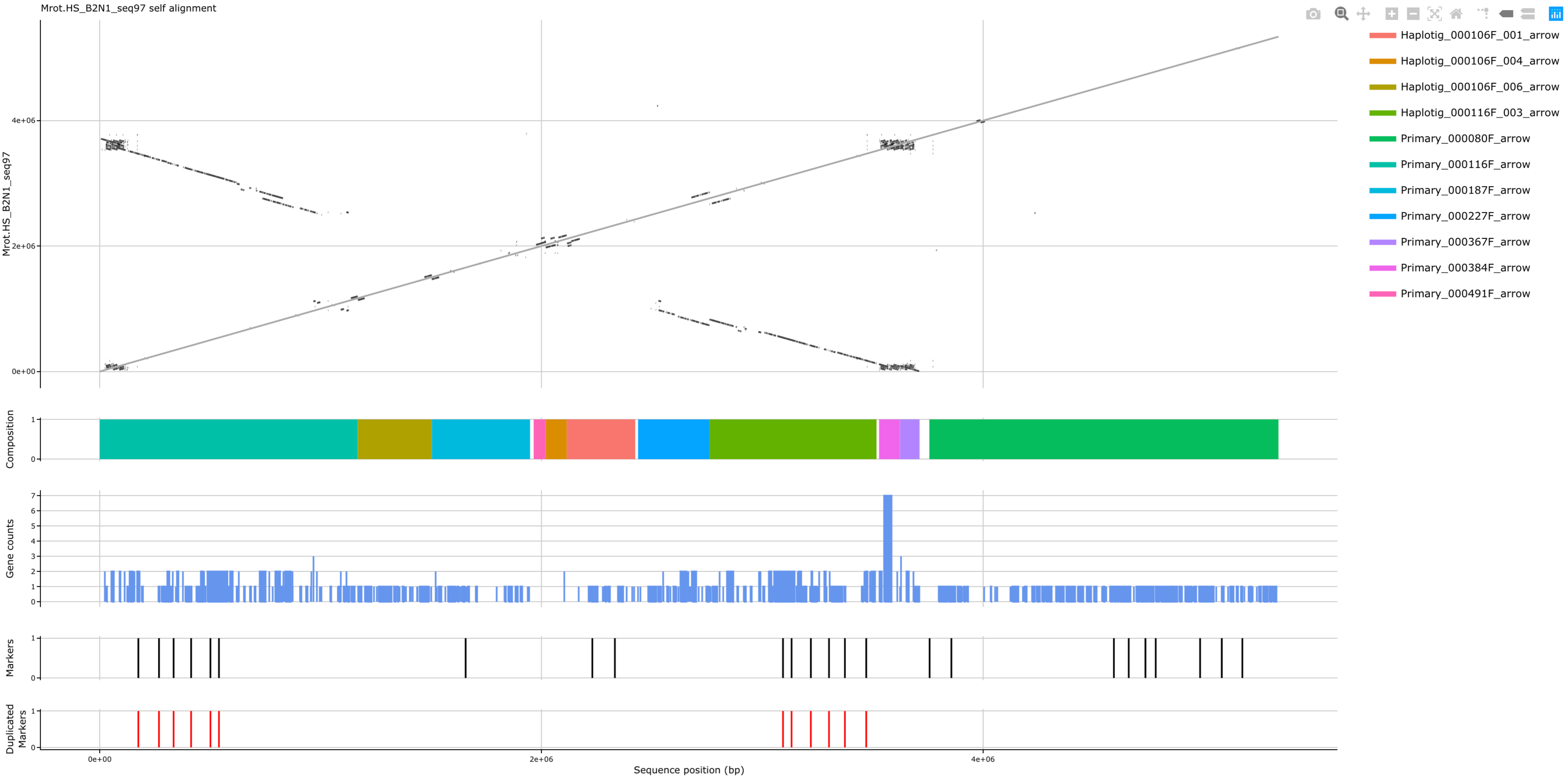

Supplement: jkac143_Supplementary_Figure_2 [file jkac143_supplementary_figure_2.pdf]

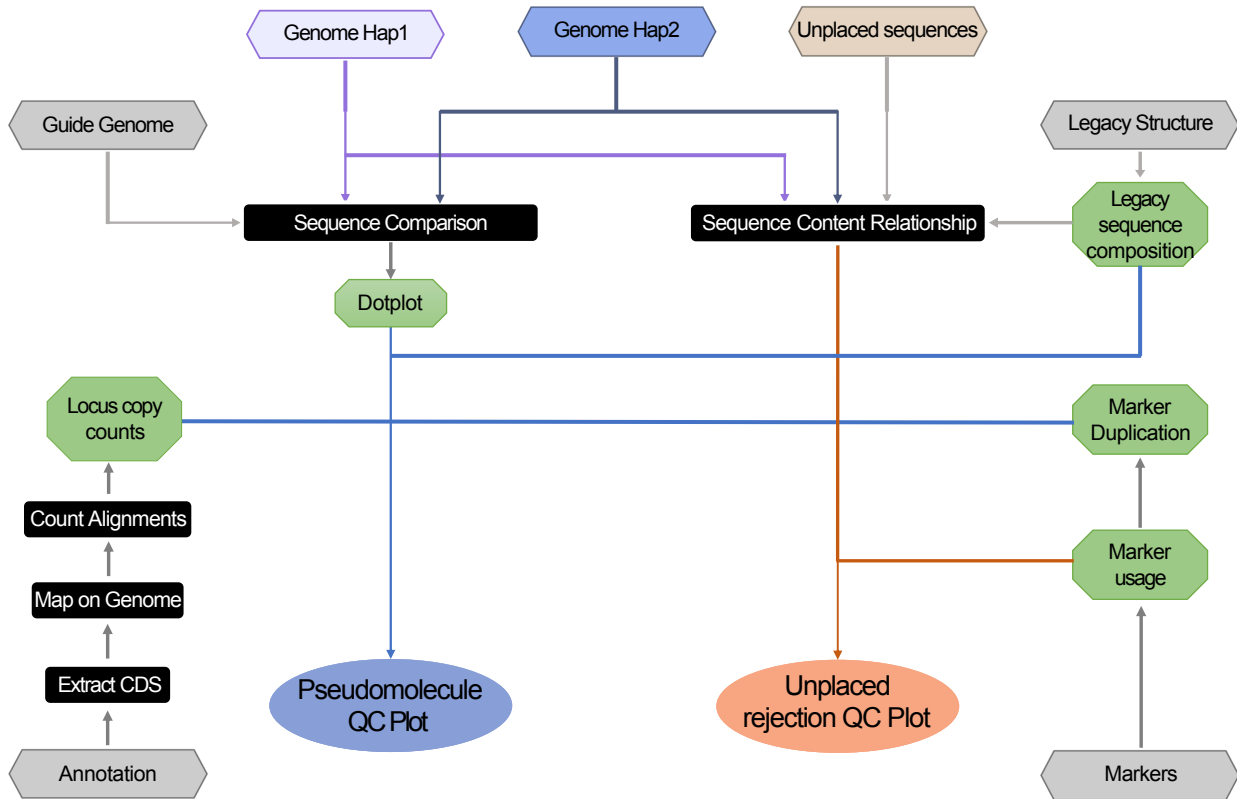

Supplement: jkac143_Supplementary_Figure_3 [file jkac143_supplementary_figure_3.pdf]

Intra-sequence duplication info report

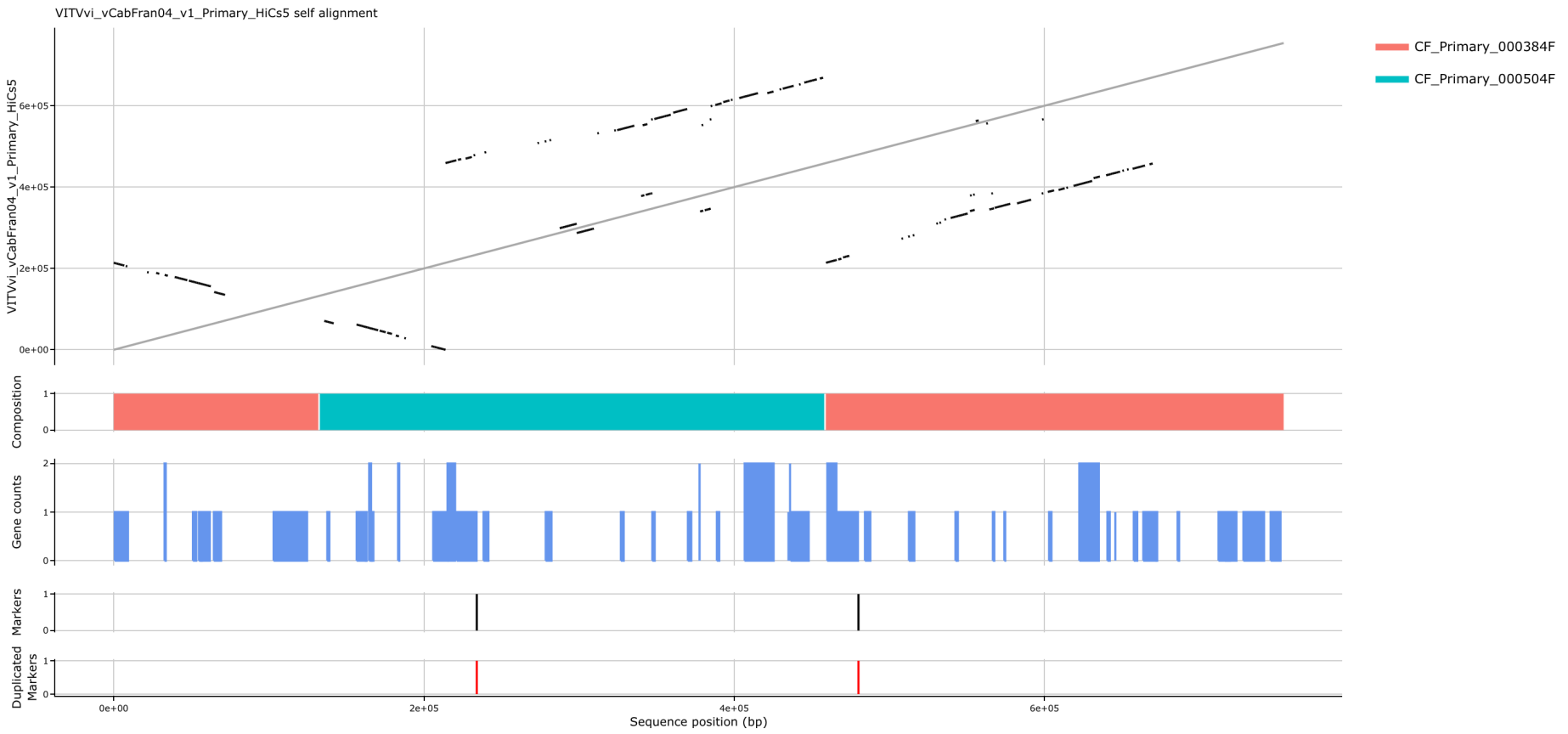

Supplement: jkac143_Supplementary_Figure_4 [file jkac143_supplementary_figure_4.pdf]

M. rotundifolia Chromosome 02 - Haplotype 1

PN40024 Chromosome 02

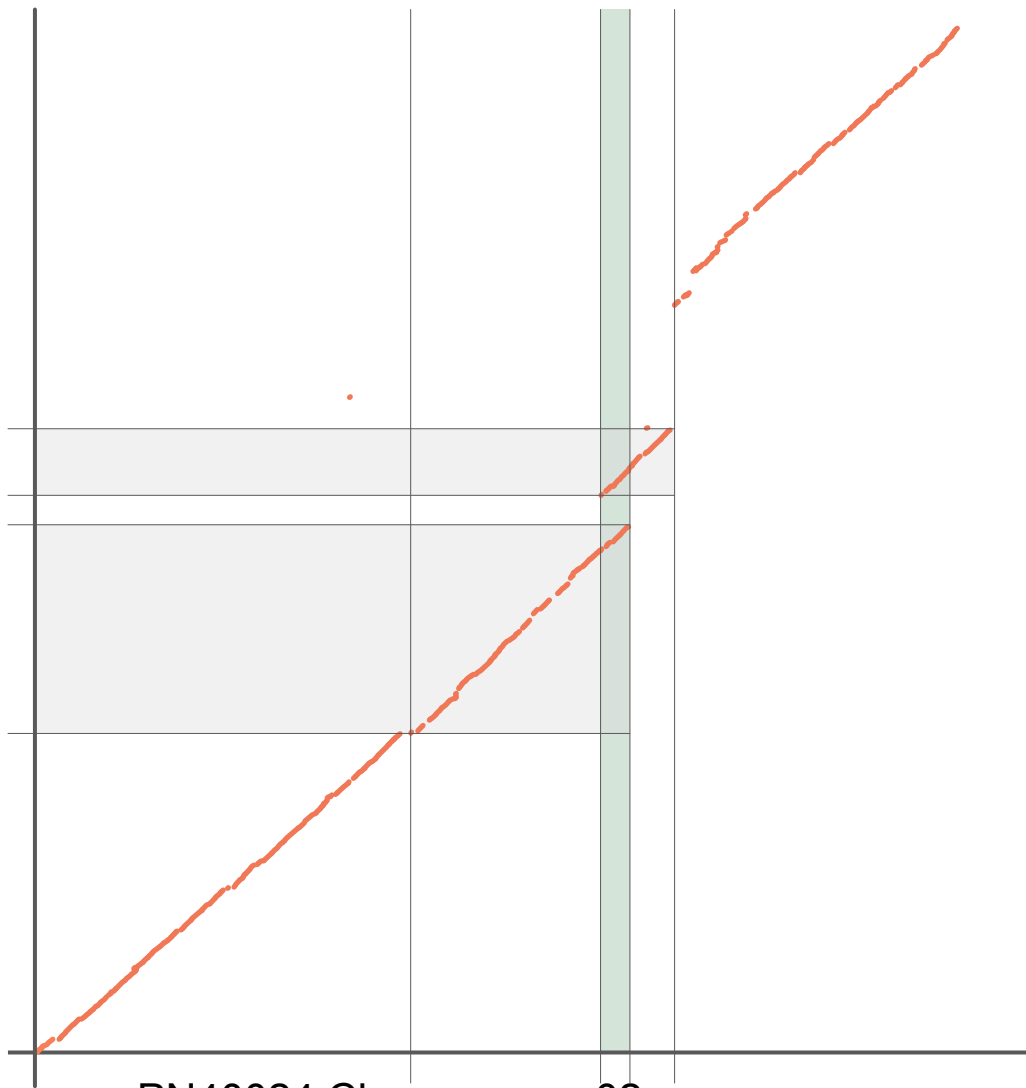

Supplement: jkac143_Supplementary_Figure_5 [file jkac143_supplementary_figure_5.pdf]

Hap1 Vs. Unplaced Vs. Hap2

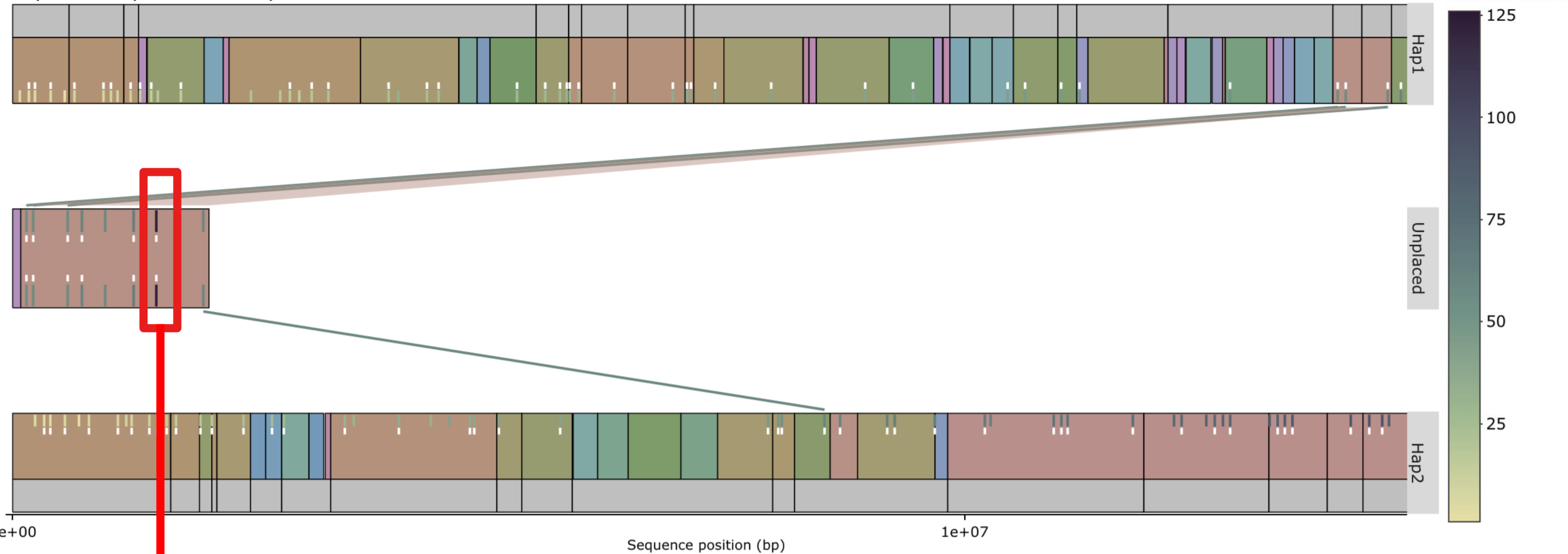

Marker ranges

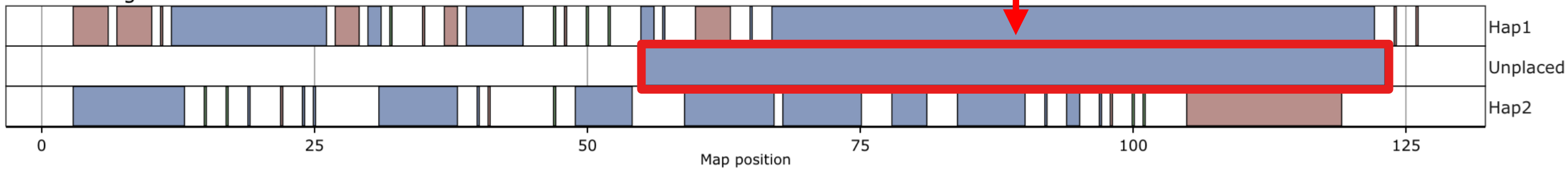

Supplement: jkac143_Supplementary_Figure_6 [file jkac143_supplementary_figure_6.pdf]

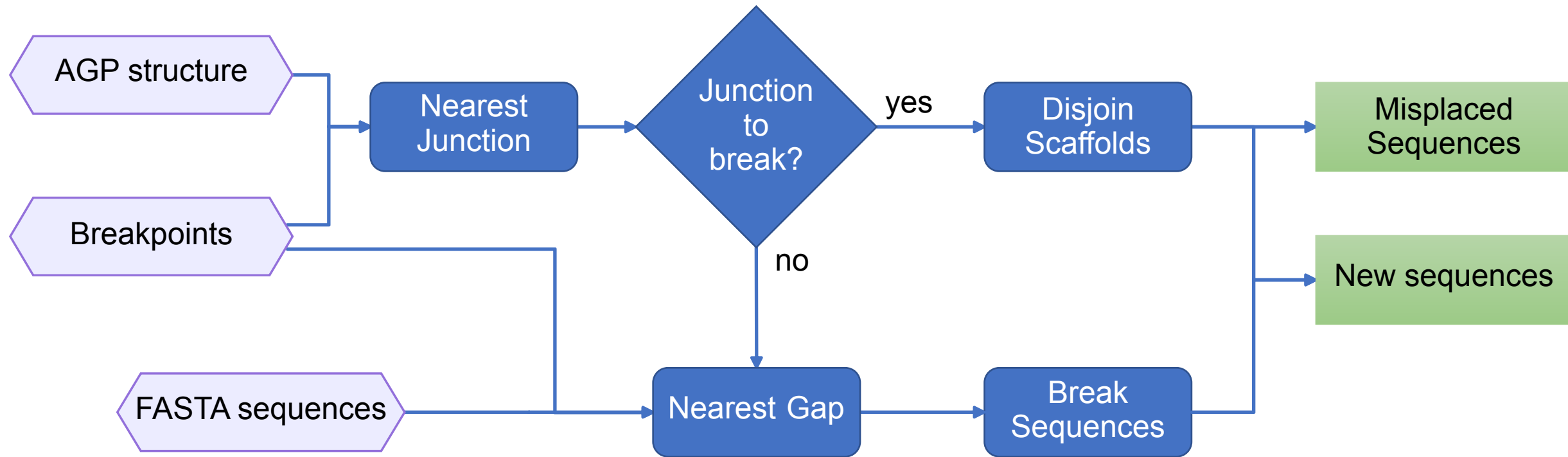

Supplement: jkac143_Supplementary_Figure_7 [file jkac143_supplementary_figure_7.pdf]

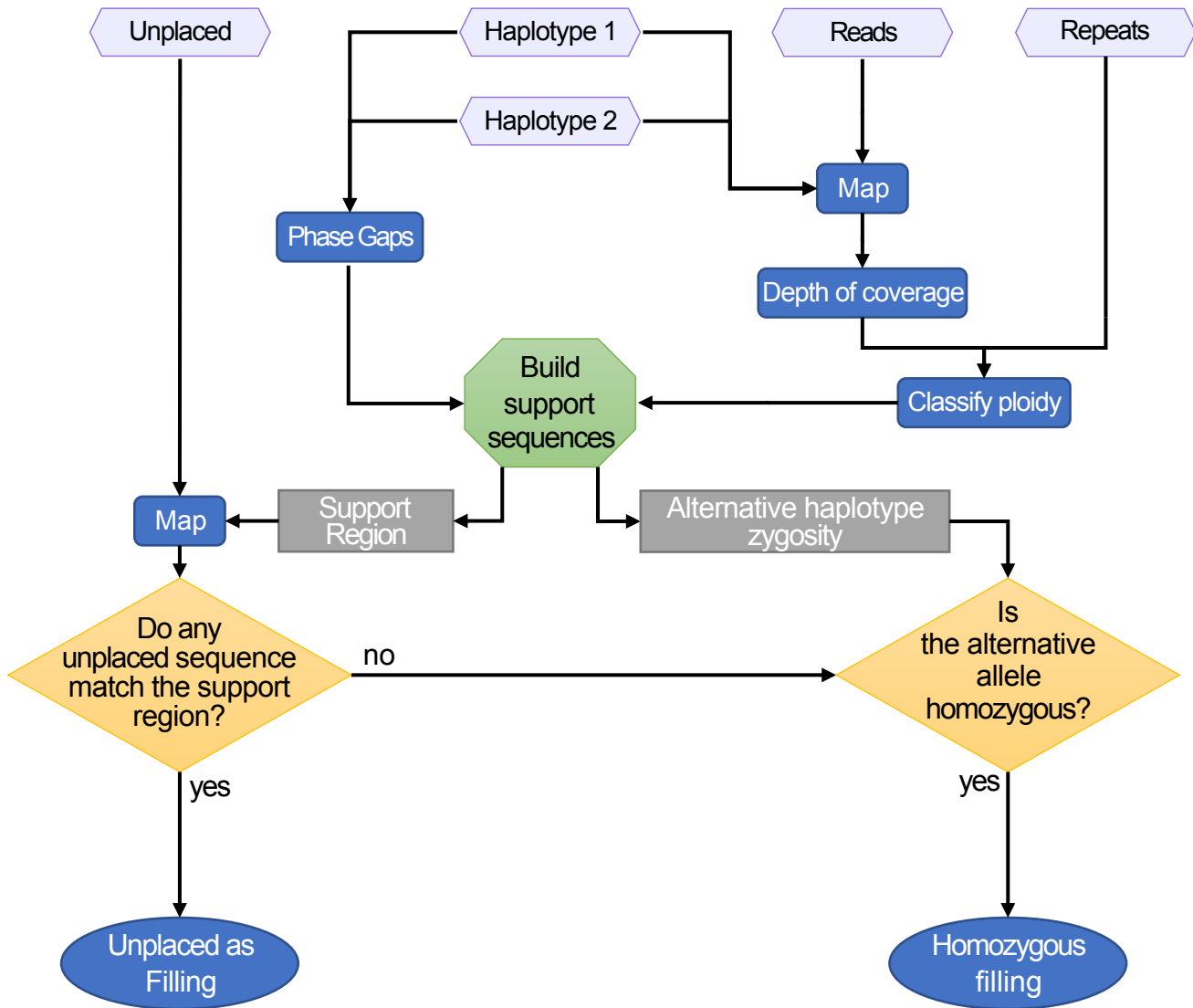

Supplement: jkac143_Supplementary_Figure_8 [file jkac143_supplementary_figure_8.pdf]

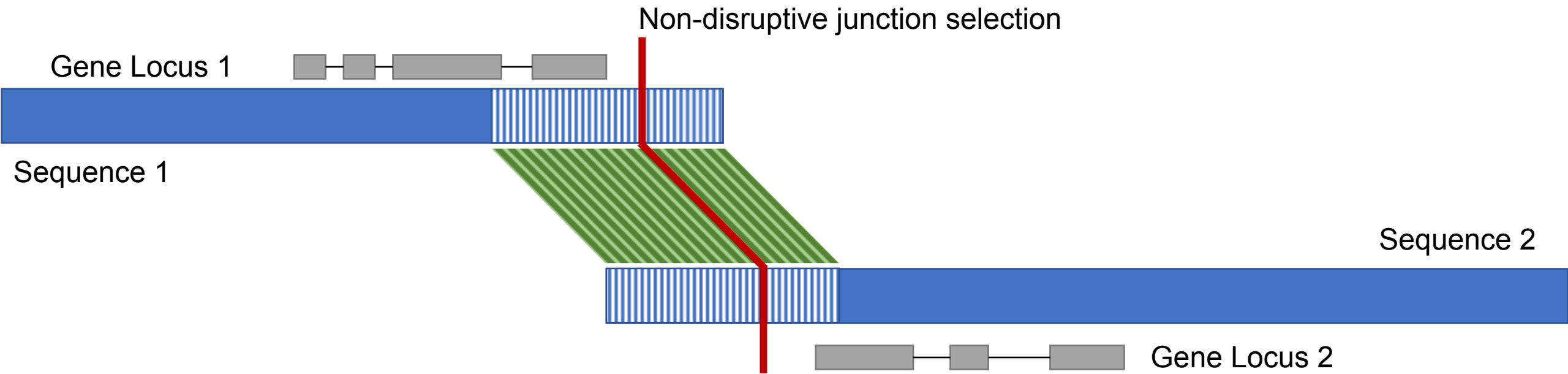

Supplement: jkac143_Supplementary_Figure_9 [file jkac143_supplementary_figure_9.pdf]

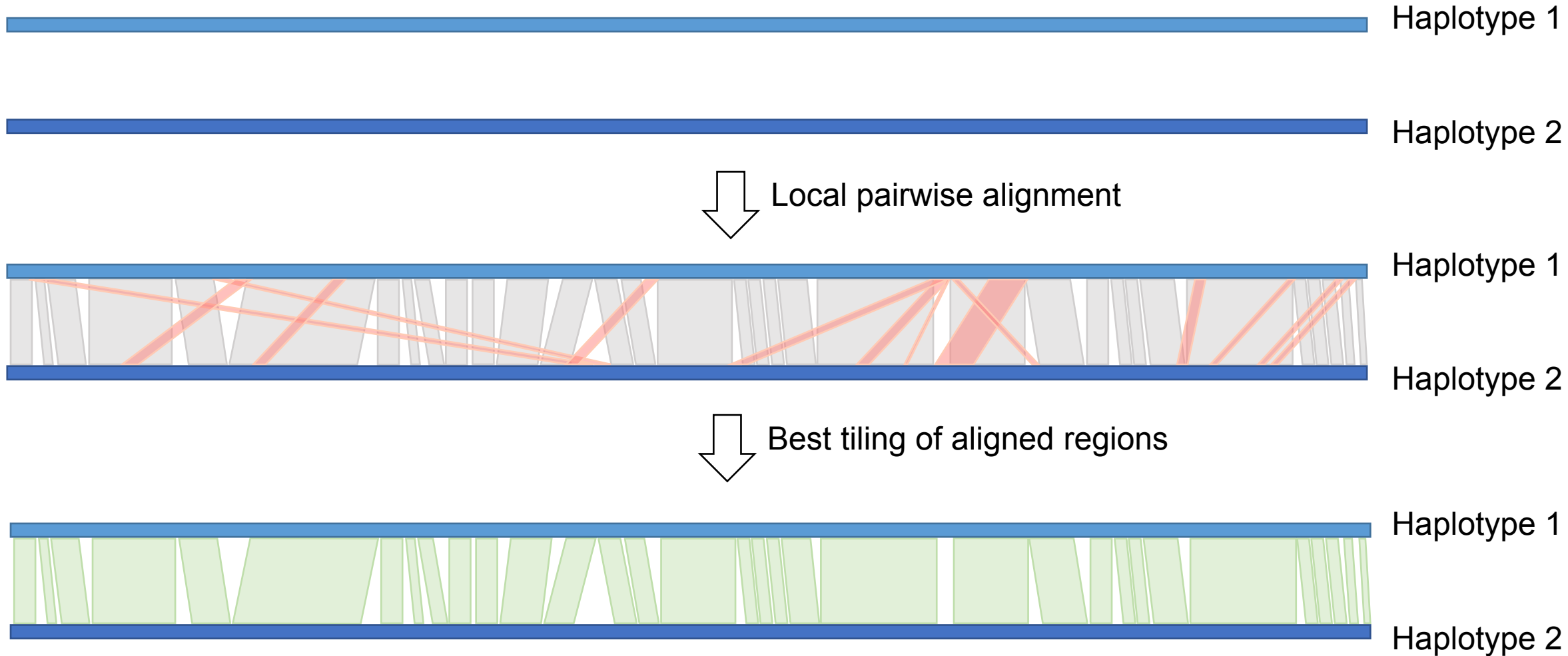

Supplement: jkac143_Supplementary_Figure_10 [file jkac143_supplementary_figure_10.pdf]

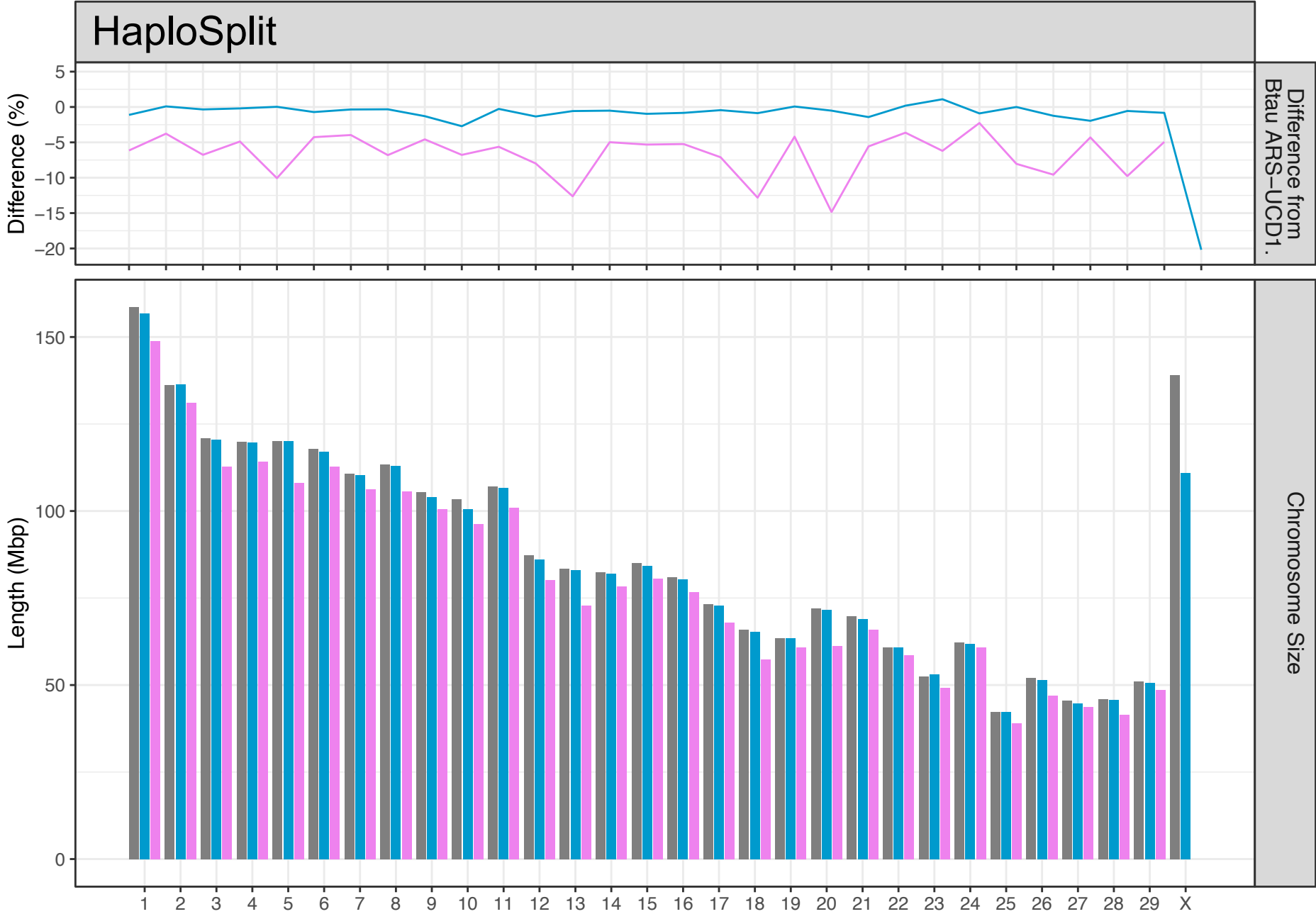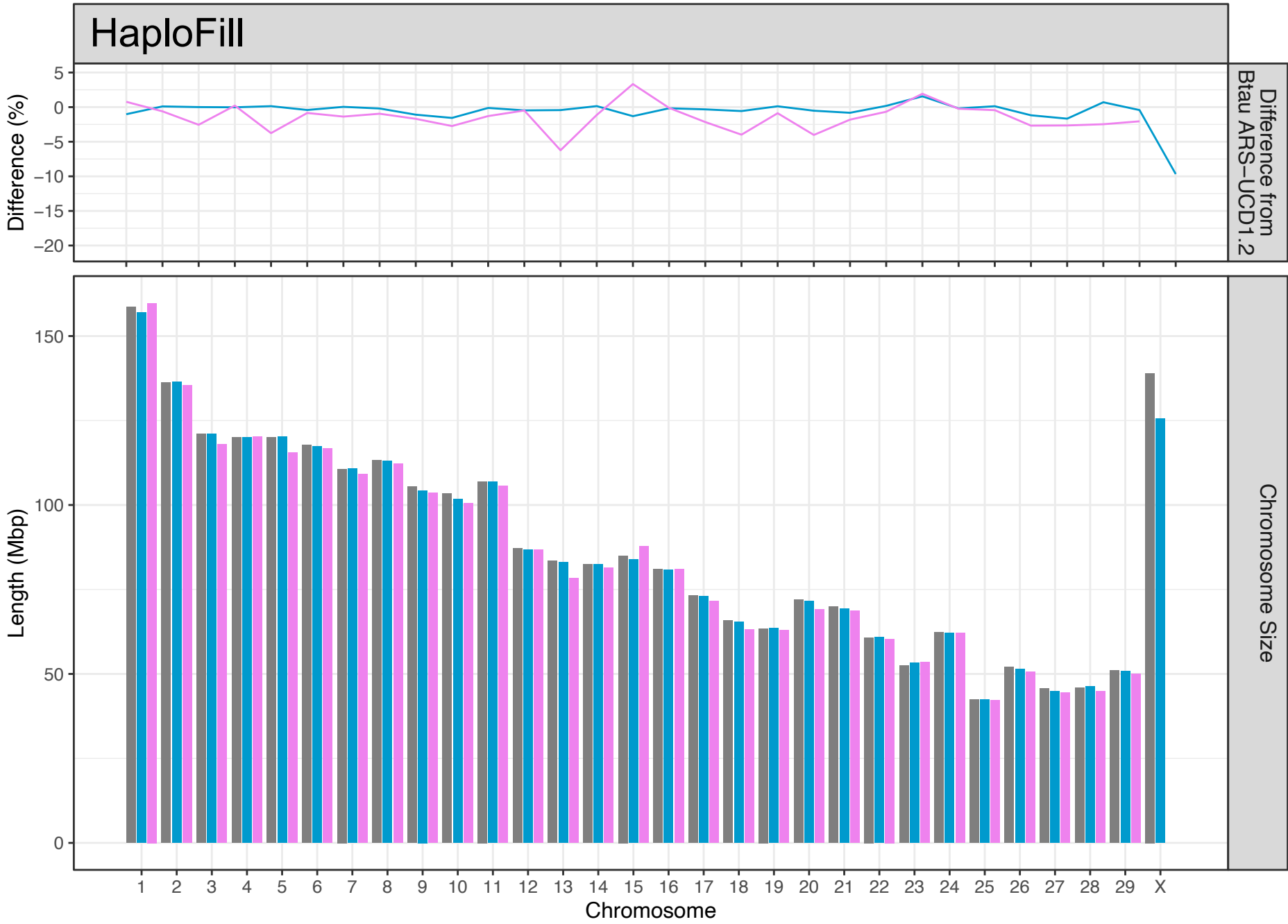

Btau ARS-UCD1.2 Hap1 Hap2

Supplement: jkac143_Supplementary_Figure_11 [file jkac143_supplementary_figure_11.pdf]

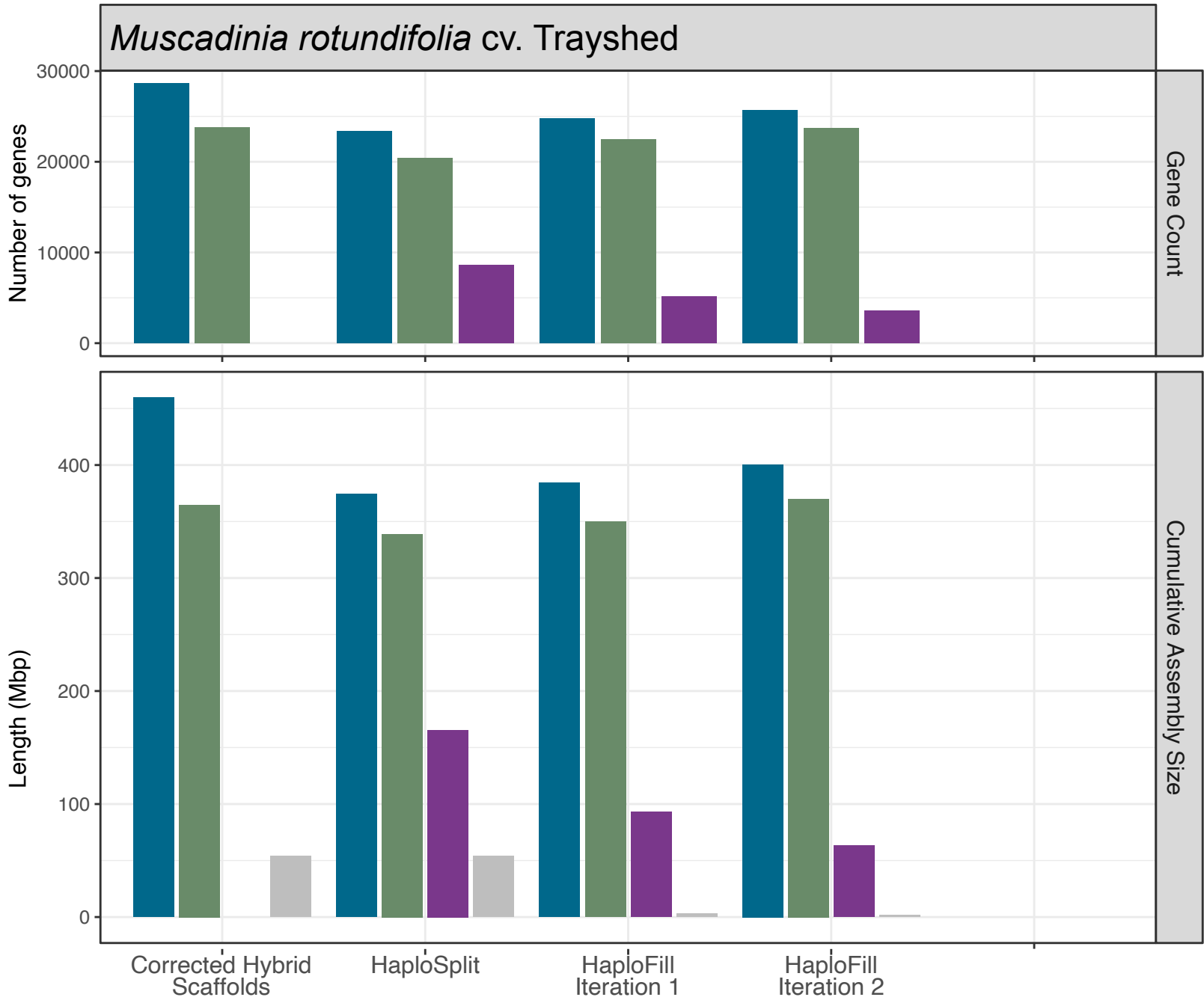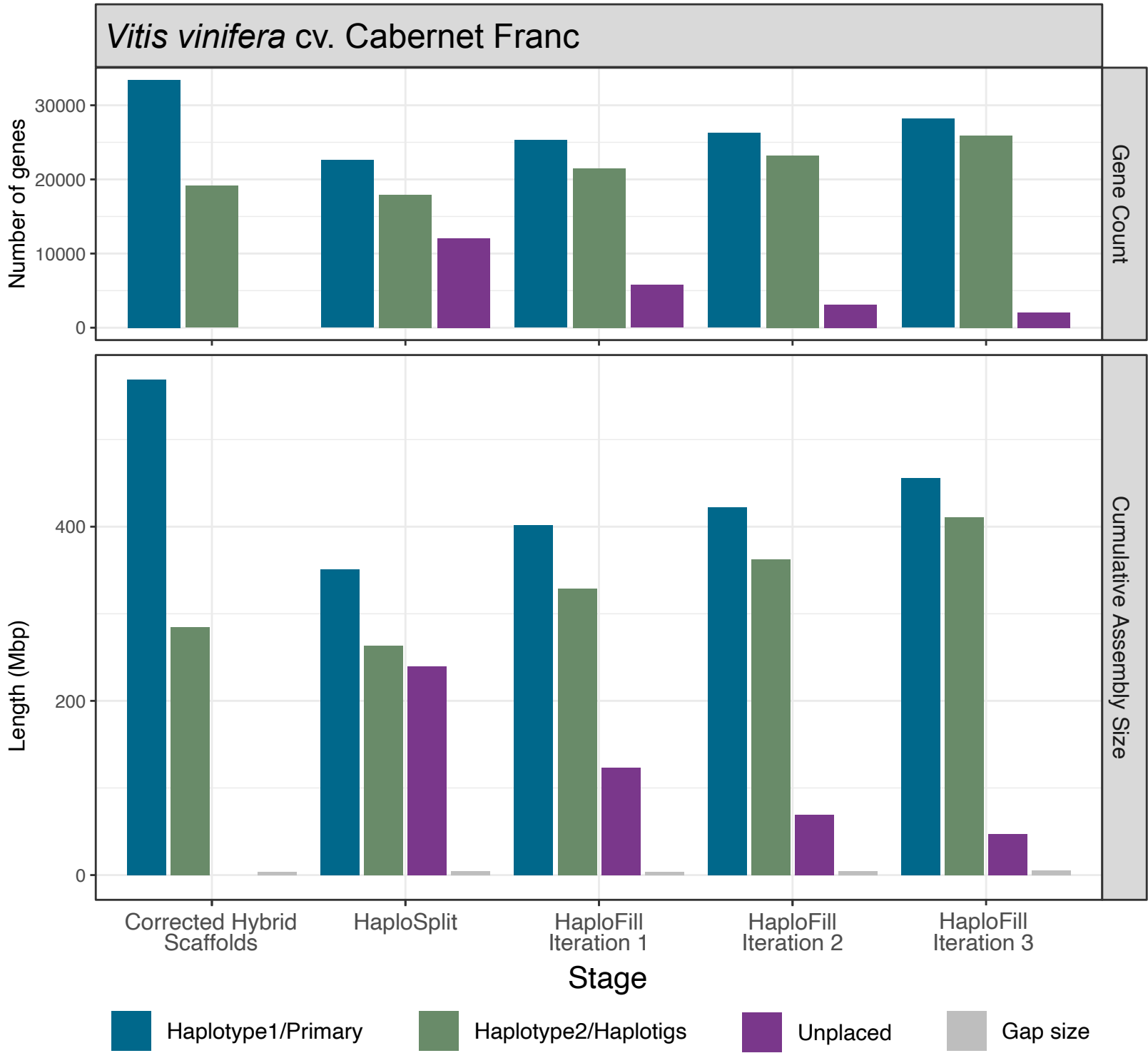

Supplement: jkac143_Supplementary_Figure_12 [file jkac143_supplementary_figure_12.pdf]

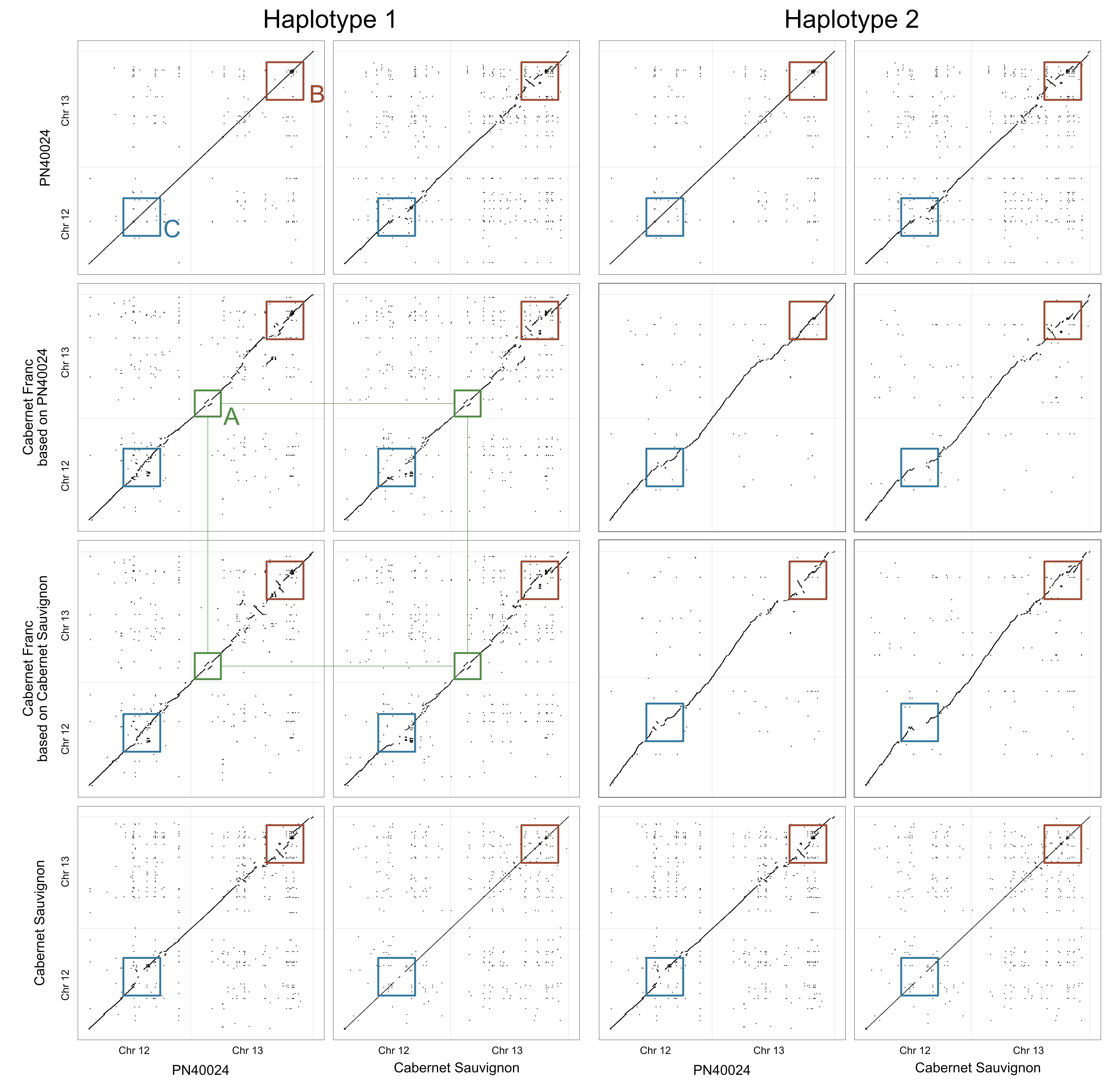

Supplement: jkac143_Supplementary_Figure_13 [file jkac143_supplementary_figure_13.jpeg]

A

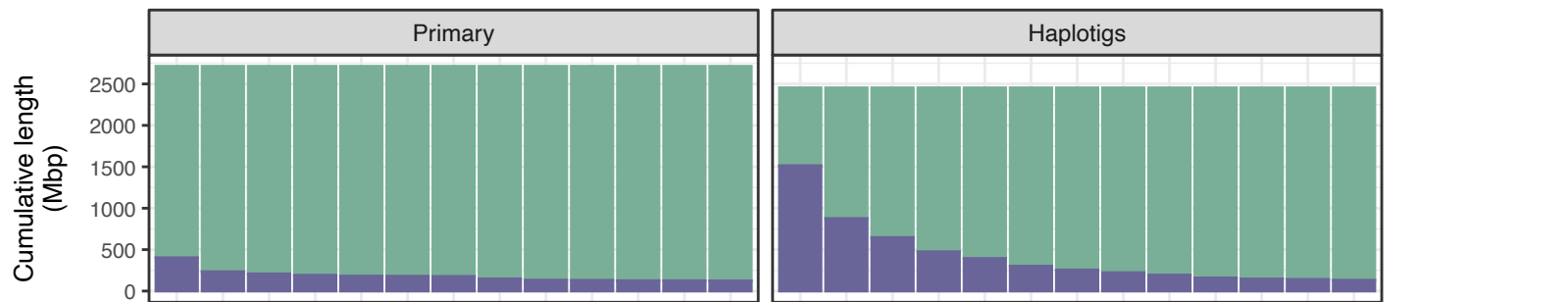

B

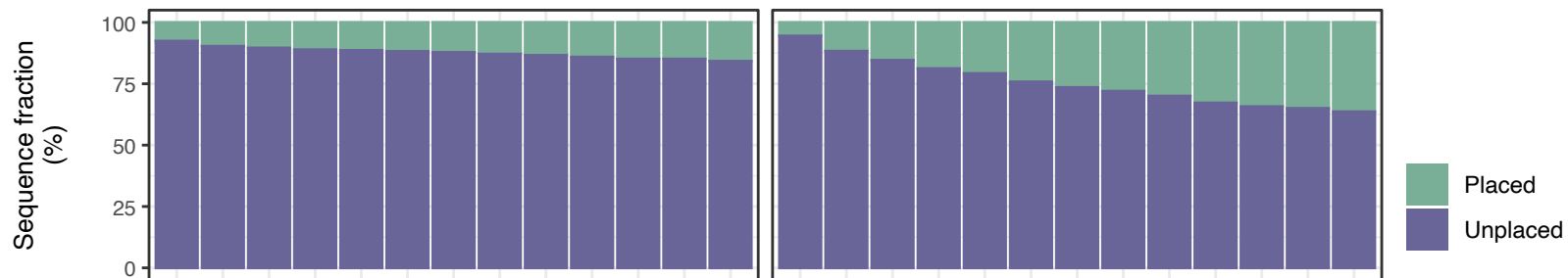

C

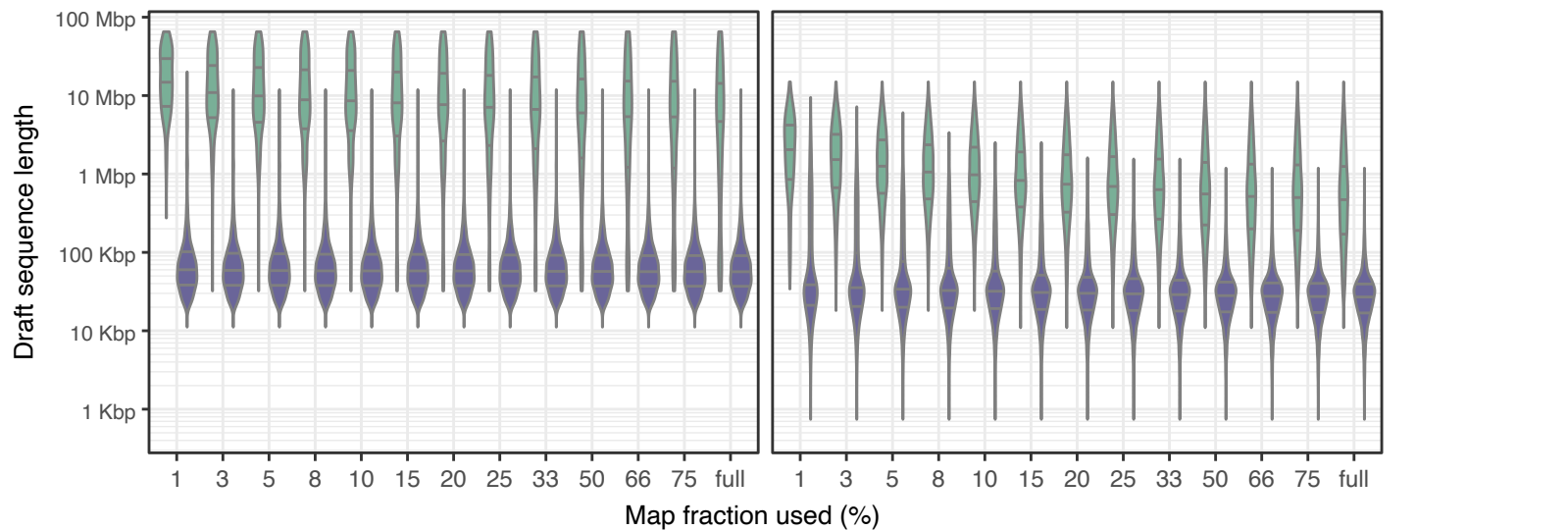

Supplement: jkac143_Supplementary_Figure_14 [file jkac143_supplementary_figure_14.pdf]
